# Gut microbiota dysbiosis in pediatric acute lymphoblastic leukemia: clinical consequences, mechanistic insights, and microbiota-targeted interventions

**DOI:** 10.3389/fped.2026.1928304

**Published:** 2026-10-06

**Authors:** Ke Chen, Qingqing Li, Yun Xing, Changqi Liu, Fei Yang

**Affiliations:** 1 Department of Clinical Nutrition, Chengdu Women’s and Children’s Central Hospital, School of Medicine, University of Electronic Science and Technology of China, Chengdu, China; 2 School of Chinese Medicine, Macau University of Science and Technology, Macau SAR, China; 3 School of Exercise and Nutritional Sciences, San Diego State University, San Diego, CA, United States; 4 Department of Virtual Reality Technology, College of Digital Technology and Design, Chongqing Polytechnic University of Electronic Technology, Chongqing, China

**Keywords:** child, leukemia, gastrointestinal microbiome, dysbiosis, probiotics

## Abstract

Gut microbiota dysbiosis has emerged as a key feature in pediatric acute lymphoblastic leukemia (ALL), driven by chemotherapy, antibiotic exposure, and treatment-related nutritional impairment. Increasing evidence indicates that disruption of microbial diversity and depletion of short-chain fatty acid–producing bacteria are closely associated with infectious complications, gastrointestinal toxicity, and potentially reduced treatment efficacy. This review synthesizes current evidence on the interplay between gut microbiota and pediatric ALL across four dimensions: clinical outcomes, treatment-related toxicity, pharmacological interactions, and prognosis. We further summarize how leukemia therapy reshapes microbial ecosystems through cytotoxic agents, antimicrobial exposure, and dietary alterations. In addition, we discuss microbiota-centered therapeutic strategies, including probiotics, prebiotics, synbiotics, postbiotics, dietary interventions, and fecal microbiota transplantation. While promising, these approaches require careful validation in pediatric oncology due to safety considerations and limited clinical trial data. Finally, we highlight methodological advances such as multi-omics integration and machine learning approaches, which may enable microbiome-based risk stratification and precision supportive care in the future. Overall, gut microbiota represents a potential modifiable factor influencing clinical outcomes in pediatric ALL.

## Highlights

1. Gut microbiota dysbiosis is evident at diagnosis in pediatric ALL patients and is further exacerbated by cytotoxic therapy, antibiotic exposure, and treatment-related nutritional impairment.
2. Dysbiosis is associated with increased infection susceptibility and chemotherapy-related toxicity, and potentially altered treatment response.
3. Emerging evidence suggests that microbiota composition may serve as a predictive biomarker for clinical outcomes in pediatric leukemia.
4. Microbiota-targeted interventions, including probiotics and dietary strategies, represent promising adjunctive approaches in supportive cancer care.

## 1 Introduction

Acute lymphoblastic leukemia (ALL) is the most common malignancy in pediatric oncology, with roughly 35–40 new cases per million children each year in industrialized regions (1, 2). Pathobiologically, ALL arises from the neoplastic expansion of primitive lymphoid precursors arrested at early differentiation stages in the bone marrow; the B-cell immunophenotype is about 80%–85% of cases (3). Over the past half-century, risk-adapted chemotherapy, targeted molecular agents, and improved supportive care have transformed ALL from a nearly uniformly fatal disease into one that is curable in most affected children, with contemporary 5-year event-free survival now exceeding 85%–90% in high-income healthcare systems (4, 5).

Yet these encouraging survival figures mask considerable treatment-related morbidity. The very intensity of modern chemotherapy required to eradicate disease produces profound myelosuppression and generalized immunocompromise, exposing patients to severe bacterial and fungal infections, febrile neutropenia, and dose-limiting mucosal toxicities (6). For the growing population of long-term survivors, persistent late effects-including metabolic disorders, excessive weight gain, cardiac dysfunction, and therapy-related second cancers-remain significant contributors to morbidity and premature mortality (7, 8). These realities have prompted investigators to look for modifiable host and environmental factors that could improve treatment tolerability, reduce toxic effects, and optimize long-term wellness.

The gut microbiota, the diverse community of bacteria, archaea, viruses, and fungi colonizing the gastrointestinal tract, has emerged as a pivotal determinant of human health and disease (9). With an estimated 10^13^–10^1^⁴ microorganisms residing in the colon, the gut microbiome encodes metabolic capabilities far exceeding those of the human genome (10). Beyond its classic roles in nutrient digestion and vitamin synthesis, the gut microbiota regulates immune system development, inflammatory homeostasis, xenobiotic metabolism, and even host behavior (11, 12). In adult oncology, accumulating evidence shows that gut microbiota composition profoundly influences carcinogenesis, treatment efficacy, and toxicities (13, 14).

The relevance of microbiota research to childhood leukemia is multifold. First, the developmental immaturity of the pediatric immune system and its intimate co-evolution with the gut microbiota suggest that early-life microbial perturbations may influence leukemia risk, consistent with Greaves' “delayed infection” theory of childhood ALL pathogenesis (15–17). Second, the profound dysbiosis induced by chemotherapy and antibiotics may contribute to the infectious complications that represent the leading cause of treatment-related morbidity and mortality in pediatric ALL (18). Third, microbiota-targeted interventions-particularly nutritional strategies-offer a potentially safe, cost-effective, and clinically feasible approach to modulating treatment outcomes in this vulnerable population (19). Dysbiosis in pediatric ALL reflects both pre-treatment ecological disturbances and therapy-induced perturbations. Baseline compositional deviations have been documented at diagnosis (20, 21), while chemotherapy and antibiotics amplify these disturbances during treatment (22, 23).

Multiple recent reviews have described gut-microbiota findings across the broader field of pediatric oncology, covering both haematological and solid tumour malignancies (24), whereas other publications specifically synthesise evidence for paediatric acute lymphoblastic leukaemia (18). This review provides a comprehensive examination of the gut microbiota in childhood leukemia, spanning pathogenesis, treatment interactions, clinical outcomes, and therapeutic modulation. We place particular emphasis on nutritional and microbiota-targeted interventions, reflecting the scope and readership of nutrition. By integrating insights from microbiology, immunology, oncology, and nutrition science, this review aims to delineate a translational roadmap for harnessing the microbiota to improve the care of children with leukemia.

The bidirectional relationship between nutrition and the microbiota deserves special attention here. Dietary components-notably dietary fibers, resistant starches, and polyphenols-serve as primary substrates for microbial fermentation, generating bioactive metabolites that modulate host immunity and metabolism (12). Conversely, microbiota composition is associated with the efficiency of nutrient extraction, vitamin biosynthesis, and energy harvest from the diet. In pediatric ALL, where nutritional status is frequently compromised by treatment-related symptoms and where microbiota diversity is profoundly disrupted, nutritional interventions represent a logical and potentially high-impact therapeutic strategy. The alignment of microbiota science with nutritional oncology creates opportunities for evidence-based dietary recommendations that extend beyond generic caloric support to targeted modulation of microbial ecosystems.

Literature Search Strategy. We conducted a comprehensive literature search using PubMed/MEDLINE, Web of Science, and Scopus databases through December 2025. Search terms included combinations of: (\"gut microbiota\” OR \"gut microbiome\” OR \"intestinal microbiota\”) AND (\"acute lymphoblastic leukemia\” OR \"ALL\”) AND (\"pediatric\” OR \"children\” OR \"childhood\”). Additional studies were identified through manual screening of reference lists from retrieved articles and recent systematic reviews. Where pediatric ALL-specific evidence was limited, we included mechanistic and preclinical studies from adult hematological malignancies, hematopoietic stem cell transplantation, or general pediatric oncology populations. The source of evidence-whether derived directly from pediatric ALL cohorts or extrapolated from related populations-is explicitly indicated throughout the manuscript.

## 2 Overview of gut microbiota development in children

### 2.1 Early-life microbiota establishment

The assembly of the human intestinal microbiota follows a remarkably stereotyped temporal pattern during infancy and early childhood, with consequences that resonate throughout immune system development and metabolic homeostasis. The longstanding paradigm of a sterile intrauterine environment has undergone substantial revision; emerging but debated evidence indicates that microbial antigen encounter may initiate prior to birth through transplacental trafficking (25). However, the possibility of contamination in low-biomass samples remains an active methodological concern, and these findings should be interpreted cautiously until replicated with stringent contamination controls (26). That said, the predominant wave of colonization unfolds during the birthing process and the immediate postnatal period, with the route of delivery serving as a paramount variable structuring pioneer community composition. Newborns delivered vaginally inherit founding populations enriched in *Lactobacillus*, *Prevotella*, and *Sneathia* taxa that mirror the maternal vaginal and fecal flora, in contrast to neonates born via cesarean delivery, whose initial communities show overrepresentation of *Staphylococcus*, *Corynebacterium,* and *Propionibacterium* lineages typical of skin surface habitats (27, 28).

Breastfeeding is the second major influence on early microbiota assembly. Human milk oligosaccharides (HMOs)-complex glycans indigestible by the infant-serve as selective growth substrates for beneficial bacteria, particularly *Bifidobacterium longum* subsp. *infantis* and *Bifidobacterium bifidum* (29). These “infant-type” bifidobacteria metabolize HMOs to produce short-chain fatty acids (SCFAs) and lactate, generating an acidic intestinal environment that suppresses pathogen colonization (29, 30). Formula-fed infants, by contrast, exhibit delayed bifidobacterial colonization and enrichment in *Clostridium difficile*, *Enterobacter cloacae*, and *Bilophila wadsworthia*-taxa associated with increased infection risk and inflammatory conditions (31, 32). Bäckhed and colleagues demonstrated that cessation of breastfeeding, rather than introduction of solid foods, forms the primary driver of microbiota maturation toward an adult-like configuration dominated by *Bacteroides*, *Roseburia*, *Clostridium*, and *Anaerostipes* (27).

Antibiotic exposure during infancy is a particularly disruptive force. Even a single course of antibiotics can profoundly alter microbiota composition, reducing *alpha* diversity and depleting beneficial taxa for months (33). The functional consequences extend beyond immediate compositional shifts: antibiotic-exposed infants demonstrate reduced SCFA production capacity, diminished colonization resistance against enteric pathogens, and altered expression of antibiotic resistance genes within the gut resistome (27, 34). These perturbations may have lasting immunological consequences, as the first 1,000 days of life constitute a critical window for microbiota-immune co-development.

### 2.2 Microbiota maturation and immune education

“Microbial community immaturity” describes a pathological developmental state wherein the intestinal microbiota fails to achieve the taxonomic complexity and functional repertoire characteristic of healthy chronological age (35). Under normal physiological conditions, the colonizing microbiota of the young child progressively enriches for genetic capabilities spanning essential amino acid anabolism, micronutrient synthesis (folate, vitamin K, and B-group vitamins), and polysaccharide catabolism-metabolic competencies that closely track the changing nutritional landscape as dietary intake diversifies (27). In parallel, the maturing mucosal immune apparatus undergoes formative education through persistent antigenic stimulation by luminal microorganisms, acquiring the capacity to distinguish harmless symbionts from potential threats and establishing appropriate tolerance-effector balances (36).

Specific commensal bacteria play non-redundant roles in immune education. Bacteroides fragilis polysaccharide A induces regulatory T cell (Treg) differentiation and interleukin-10 (IL-10) production, promoting mucosal tolerance (37). Segmented filamentous bacteria (SFB) stimulate the differentiation of T helper 17 (Th17) cells. These are essential for mucosal defense against fungal and bacterial pathogens (38). SCFA-producing bacteria-particularly *Faecalibacterium prausnitzii*, *Roseburia* species, and *Eubacterium* rectale-promote Treg development and maintain intestinal barrier integrity through histone deacetylase (HDAC) inhibition and G-protein-coupled receptor (GPR) signaling (39, 40). Disruption of these immunomodulatory dialogues during the critical neonatal window may predispose to immune-mediated diseases, including allergy, autoimmunity, and potentially malignancy.

### 2.3 Link to leukemia susceptibility

The hypothesis that gut microbiota immaturity contributes to childhood leukemia risk draws substantial support from epidemiological and experimental evidence. Greaves' delayed infection theory posits that ALL arises through a two-step process: an initiating genetic lesion (frequently ETV6-RUNX1 fusion) acquired *in utero*, followed by a secondary transforming event triggered by aberrant immune responses to common childhood infections in genetically predisposed individuals (15, 41). Children with limited early-life microbial exposure-due to excessive hygiene, lack of sibling contact, or absence of daycare attendance-may fail to develop properly “primed” immune systems, rendering them susceptible to dysregulated inflammatory responses upon later infection that contribute leukemic transformation (16, 42).

Kameri and colleagues recently reviewed evidence linking microbiota-targeting risk factors to childhood ALL, identifying cesarean delivery, lack of breastfeeding, and early-life antibiotic use as exposures associated with both microbiota perturbation and increased leukemia risk (16). Daycare attendance during infancy-which facilitates microbial transmission and immune stimulation-has been associated with a 32% reduction in ALL risk [odds ratio [OR] 0.68, 95% confidence interval [CI] 0.48–0.95] in Danish cohort studies (43). Animal models suggest mechanistic support: ETV6-RUNX1-transgenic mice housed under specific-pathogen-free (SPF) conditions develop leukemia only when exposed to common pathogens, suggesting that microbial exposure is required for malignant transformation. Conversely, germ-free conditions prevent leukemogenesis in these models, indicating that the absence of microbial stimulation- or the lack of inflammatory signals derived therefrom- blocks clonal evolution (41, 44). These converging lines of evidence suggest that the gut microbiota is a modifiable determinant of leukemia susceptibility, opening avenues for primary prevention strategies.

### 2.4 Factors shaping pediatric microbiota

Beyond delivery mode, feeding practices, and antibiotics, multiple environmental and genetic factors sculpt the pediatric gut microbiota. Dietary patterns during weaning and early childhood-including fiber intake, fermented food consumption, and exposure to diverse food antigens-contribute the expansion of polysaccharide-degrading taxa and SCFA-producing communities (45). Geographic location and living environment modulate microbial diversity, with children raised in rural settings or in proximity to livestock displaying richer, more diverse microbiota than their urban counterparts (46). Host genetics, including polymorphisms in genes encoding pattern recognition receptors (e.g., NOD2, TLRs) and fucosyltransferase enzymes, also influence microbiota composition and stability (47).

Understanding these determinants is essential for contextualizing microbiota differences observed between leukemia patients and healthy controls, and for designing targeted interventions. Recognizing the plasticity of the pediatric microbiota during the first years of life highlights the potential for early-life preventive strategies that could modulate leukemia risk before disease initiation-an area of considerable public health significance given the rising incidence of childhood ALL in many countries. The identification of modifiable risk factors such as breastfeeding, antibiotic stewardship, and daycare attendance offers concrete targets for population-level interventions aimed at supporting healthy microbiota development during this critical window.

## 3 Gut microbiota alterations in childhood leukemia

### 3.1 Baseline dysbiosis before treatment

Emerging data challenge the assumption that microbiota perturbations in pediatric ALL arise exclusively as treatment sequelae, instead pointing to measurable compositional deviations present at disease presentation (18). Rajagopala and coworkers reported that among 32 newly diagnosed ALL patients, both taxonomic diversity and community richness were substantially attenuated relative to 25 healthy sibling comparator subjects, with these differences detectable before cytotoxic therapy administration (20). Whether such pre-treatment ecological disturbances represent causally implicated factors in leukemogenesis, downstream consequences of disease-mediated changes in host inflammatory tone, metabolism, or immunological status, or coincidental associations remains to be definitively established.

Bai and colleagues further demonstrated that ALL patients not exposed to antibiotics exhibited significant differences in *alpha-*diversity compared to healthy controls, as well as distinct *beta*-diversity patterns regardless of antibiotic use (21). Meta-analytic evidence (48) suggests enrichment in *Bacteroides* species at diagnosis, though individual study findings remain heterogeneous (21), and depletion of beneficial Firmicutes such as *Faecalibacterium* and *Roseburia.* The functional implications of these shifts extend beyond taxonomy: a recent shotgun metagenomic study of treatment-naïve ALL patients revealed heightened bile acid-transforming potential linked to *Clostridium scindens* and *Ruthenibacterium* lactatiformans, alongside reduced butyrate- and lactate-producing capacity compared to healthy controls (49).

The enrichment of *Bacteroides* at diagnosis is particularly intriguing. While *Bacteroides* species are normal constituents of the healthy gut microbiota, their overrepresentation may reflect altered intestinal ecology or impaired competitive exclusion by other commensals. Compositional analyses have identified variable signatures of baseline dysbiosis, with some studies reporting enrichment in Bacteroides species (48) while others find no significant Bacteroides enrichment (20, 21). Increased inter-individual variability in microbiota composition among newly diagnosed ALL patients-exceeding that observed in healthy children-further underscores the ecological instability characteristic of the pre-treatment state (20, 21). Whether these baseline alterations represent causative factors, disease biomarkers, or consequences of leukemia-related metabolic changes remains an active area of investigation.

### 3.2 Microbiota dynamics during chemotherapy

Cytotoxic chemotherapy inflicts severe collateral damage upon the intestinal microbial ecosystem, amplifying any pre-existing community disturbances. In the most extensive longitudinal pediatric ALL microbiota investigation reported to date, Hakim and coworkers prospectively tracked 199 children through sequential therapeutic phases-induction, consolidation, and reinduction-documenting significant erosion of microbial diversity following both induction and reinduction cycles compared to pre-chemotherapy reference samples (22). Taxonomic restructuring was pronounced and directionally coherent: populations belonging to the phylum Bacteroidetes, the genus *Faecalibacterium*, the family Ruminococcaceae, phylum Actinobacteria, and phylum Verrucomicrobia exhibited marked contraction, whereas *Clostridiaceae*, *Streptococcaceae*, *Lactobacillaceae*, *Enterococcaceae*, and certain Firmicutes lineages underwent proportional expansion (18, 22).

Wang and colleagues employed shotgun metagenomics to characterize acute microbial changes during induction chemotherapy in 49 children with ALL, collecting samples at treatment initiation (T0), day 7 (T1), and day 33 (T2) (23). They observed progressive decreases in microbial richness and evenness, with dramatic expansion of opportunistic pathogens including *Escherichia coli* and *Klebsiella pneumoniae* and parallel depletion of beneficial species such as *Bifidobacterium longum* and *Akkermansia muciniphila.* The expansion of Proteobacteria-a phylum encompassing numerous Gram-negative pathogens-emerged as a consistent signature of chemotherapy-induced dysbiosis, potentially driven by intestinal inflammation and the facultative anaerobic metabolism that enables these organisms to thrive in disrupted ecosystems (23, 50). In adult bone marrow transplant patients, Proteobacteria expansion has been documented following chemotherapy (50). Whether this pattern is conserved in pediatric ALL requires direct validation, as adult and pediatric chemotherapy regimens differ substantially in intensity and supportive care protocols.

Functional metagenomic analyses revealed profound metabolic pathway alterations during chemotherapy, including perturbations in pyruvate fermentation to acetate and lactate, and disruption of assimilatory sulfate reduction pathways (23). The clinical significance of these functional shifts is underscored by the finding that baseline *B. longum* abundance predicted infectious complications during the first month of chemotherapy with an area under the receiver operating characteristic curve (AUC) of 0.720 (23). The most dramatic microbiota changes occurred within the first month of chemotherapy-a window of particular vulnerability when patients are severely immunosuppressed and at peak risk for infectious complications.

### 3.3 Long-term alterations in survivors

The impact of ALL therapy on the gut microbiota extends well beyond treatment completion. Multiple studies have documented persistent dysbiosis in long-term survivors, with potential implications for metabolic and immunological health. In a study by Rajagopala and colleagues, although gut microbial diversity tended to stabilize one year post-chemotherapy, species abundances remained significantly altered, with continued elevation of mucolytic Gram-positive anaerobes including *Ruminococcus gnavus* and *Ruminococcus torques* (20). These species degrade mucin glycoproteins within the protective intestinal mucus layer, thereby compromising epithelial barrier integrity and contributing to chronic low-grade inflammation.

Thomas and colleagues compared the gut microbiota of ALL survivors (at least one year after treatment completion) with healthy siblings, detecting depletion of *Lachnospiraceae* and *Ruminococcaceae* families-including the butyrate producer *Faecalibacterium-*despite no statistically significant differences in *alpha* or *beta* diversity (51). Bhutta and colleagues further observed that ALL survivors (median 24 months post-treatment) exhibited significantly lower Pielou's evenness and distinct microbial composition compared to healthy siblings (52). These findings suggest that while broad diversity metrics may partially recover, specific taxonomic and functional deficits persist.

The most compelling evidence for long-term dysbiosis comes from Chua and colleagues, who studied adult survivors of pediatric ALL who had completed therapy at least five years prior (53). Compared to healthy controls, these survivors showed reduced microbial diversity, enrichment of *Actinobacteri*a, and striking depletion of *Faecalibacterium*. these microbiota alterations correlated with increased T-cell activation and markers of chronic systemic inflammation-providing a plausible mechanistic link between treatment-induced dysbiosis and the elevated risk of metabolic syndrome, cardiovascular disease, and secondary immune dysfunction observed in ALL survivors (53, 54).

### 3.4 Functional and multi-omics insights

Recent methodological advances have enabled unprecedented resolution in characterizing microbiota functional capacity in childhood ALL. Shotgun metagenomics-reviewed in detail in Section 8-has revealed strain-level compositional differences and metabolic pathway alterations that remain invisible to 16S rRNA gene sequencing (23, 49). Beyond metagenomics, metabolomic profiling has demonstrated altered fecal concentrations of SCFAs, amino acid derivatives, and bile acid metabolites in ALL patients compared to controls (55).

The integration of multi-omic datasets has begun to yield systems-level insights. For instance, the combination of shotgun metagenomics with fecal metabolomics has confirmed that depletion of SCFA-producing taxa in ALL patients translates into functionally meaningful reductions in butyrate, propionate, and acetate concentrations (55). These metabolites, detailed in Section 4, are critical for intestinal barrier maintenance, immune regulation, and epigenetic modulation-suggesting that microbiota-targeted restoration of SCFA production could offer therapeutic benefit. The emergence of integrated multi-omics platforms is a transformative opportunity to move beyond descriptive compositional studies toward mechanistic understanding of microbiota-leukemia interactions. Such integrative approaches may ultimately enable the identification of causal microbial drivers of treatment outcomes, informing the rational design of microbiota-targeted nutritional interventions for this vulnerable patient population (Figure 1).

**Figure 1:**
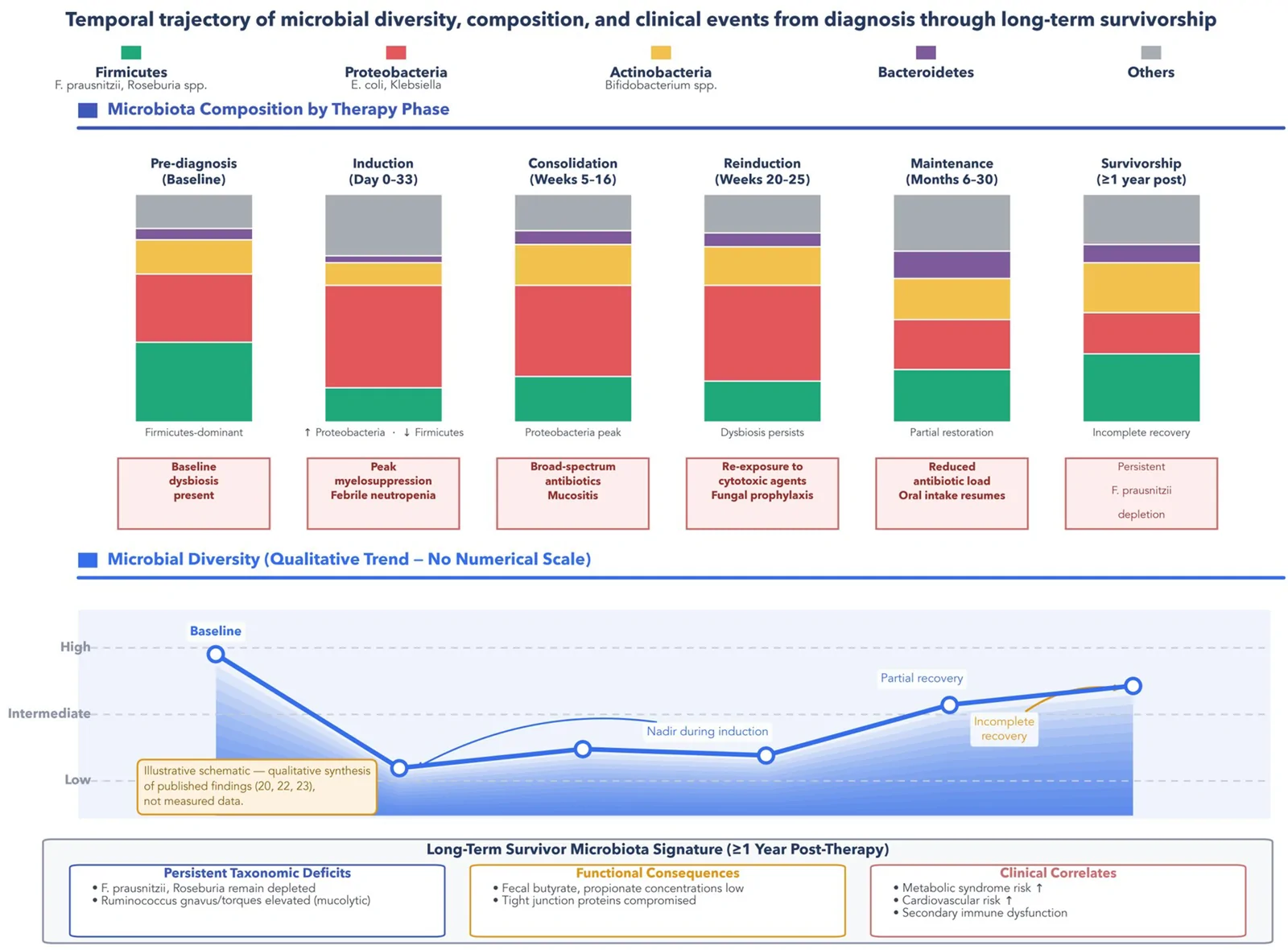
Temporal trajectory of microbial diversity, composition, and clinical events from diagnosis through long-term survivorship.

Beyond bacteria, the gut ecosystem encompasses viral communities (virome) and fungal populations (mycobiome) that interact with bacterial microbiota and host immunity. Emerging evidence in adult oncology suggests that fungal dysbiosis-particularly Candida overgrowth-may contribute to mucosal barrier dysfunction and invasive infection during chemotherapy. In pediatric ALL, where candidiasis is a recognized complication, the mycobiome represents an understudied dimension of the gut ecosystem. Similarly, bacteriophage dynamics within the gut virome may influence bacterial community structure and antibiotic resistance gene transfer. However, pediatric ALL-specific data on the virome and mycobiome are currently lacking, and their clinical relevance in this population remains speculative.

This figure is a qualitative schematic synthesizing published data (20, 22, 23) and is not derived from a single cohort or a formal pooled analysis; no quantitative values are displayed. Stacked bars depict the direction and approximate magnitude of phylum-level shifts from pre-diagnosis through survivorship based on pooled published findings: Firmicutes depletion and Proteobacteria expansion peak during intensive therapy, followed by partial and incomplete restoration. The diversity trajectory (blue line) illustrates the qualitative pattern of diversity decline during intensive therapy and incomplete recovery in long-term survivors; absolute Shannon index values vary substantially across studies with different sequencing platforms and bioinformatic pipelines and are therefore not displayed numerically. Clinical events are annotated at representative therapy phases. The bottom panel summarizes persistent taxonomic deficits, functional consequences, and clinical correlates reported in long-term survivors (≥1 year post-therapy).

Given the substantial heterogeneity across existing pediatric ALL microbiome studies—including variation in cohort size, treatment phase, sequencing methodology, and antibiotic exposure—we have provided a comprehensive summary table (Table 1) to facilitate transparent evaluation of the evidence base and to clearly distinguish findings derived directly from pediatric ALL cohorts from those extrapolated from related populations.

**Table 1:** Summary of major pediatric acute lymphoblastic leukemia (ALL) microbiome studies.

Study	Cohort(n)	Treatment Phase	Sequencing Approach	Antibiotic Data	Main Findings	Clinical Endpoints
Hakim et al., 2018 (22)	199	Induction, consolidation, reinduction	16S (V1-V3)	Yes	Significant diversity erosion post-induction and reinduction. Bacteroidetes, Faecalibacterium, Ruminococcaceae, Actinobacteria, and Verrucomicrobia contracted; Clostridiaceae, Streptococcaceae, Lactobacillaceae, Enterococcaceae, and Firmicutes expanded.	Febrile neutropenia (OR 2.8 for Enterococcaceae ≥30%), diarrheal illness, bloodstream infections
Wang et al., 2024 (23)	49	Induction (T0, T1, T2)	Shotgun	NR	Progressive decrease in richness and evenness. Expansion of Escherichia coli and Klebsiella pneumoniae; depletion of Bifidobacterium longum and Akkermansia muciniphila. Proteobacteria expansion consistent.	Infectious complications (B. longum AUC = 0.720, 95% CI 0.563–0.877, *p* = 0.019)
Muratore et al., 2025 (49)	NR	Treatment-naïve	Shotgun	NR	Distinct functional and compositional properties in treatment-naïve ALL. Elevated bile acid-transforming potential (Clostridium scindens, Ruthenibacterium lactatiformans). Reduced butyrate- and lactate-producing capacity.	None (cross-sectional)
Rajagopala et al., 2020 (20)	32	Pre-treatment, post-treatment (1 yr)	16S (V4)	Partial	Baseline diversity and richness attenuated vs. healthy siblings. Species abundances remained altered at 1 yr post-chemotherapy. Elevation of mucolytic Ruminococcus gnavus and R. torques.	Long-term compositional changes
Sørum et al., 2025 (56)	NR	Induction	16S	NR	Changes in gut microbiota predict neutropenia after induction treatment. Baseline Ruminococcaceae and Lachnospiraceae predicted prolonged neutropenia; Enterococcus dominance associated with delayed neutrophil recovery.	Prolonged neutropenia, neutrophil recovery time
Bhuta et al., 2021 (52)	∼30	Post-treatment (median 24 mo)	16S	NR	Significantly lower Pielou's evenness and distinct microbial composition compared to healthy siblings. Persistent dysbiosis in long-term survivors.	None (observational)
Chua et al., 2017 (53)	20+	Adult survivors (≥5 yr post-therapy)	16S	NR	Reduced microbial diversity, enrichment of Actinobacteria, striking depletion of Faecalibacterium. Correlated with increased T-cell activation and chronic systemic inflammation markers.	Metabolic markers, inflammatory indices
Bai et al., 2017 (21)	45+	Pre-treatment	16S	Yes	ALL patients exhibited significant alpha-diversity differences vs. healthy controls regardless of antibiotic use. Distinct beta-diversity patterns. Bacteroidales and Enterococcaceae as possible biomarkers.	None (cross-sectional)
Chen et al., 2022 (57)	NR	Pre- and post-chemotherapy	16S	NR	Intestinal flora changes before and after chemotherapy in children with ALL. Decreased beneficial bacteria and increased conditional pathogens post-chemotherapy.	None (descriptive)
Biagi et al., 2015 (58)^a^	Pediatric	HSCT	16S	Yes	Gut microbiota trajectory during pediatric HSCT. EN promoted faster recovery of diversity and SCFA production vs. PN.	Bloodstream infections, aGVHD
D'Amico et al., 2022 (59)^a^	Pediatric hematologic malignancies	Induction	16S	NR	Oral lactoferrin supplementation during induction promoted gut microbiome eubiosis. Increased beneficial taxa and reduced pathogen colonization.	Microbiome composition (intervention trial)
MacDonald et al., 2023 (60)	Pediatric leukemia/lymphoma	NR	Shotgun	Yes	Gastrointestinal antibiotic resistome characterization. Enrichment of antibiotic resistance genes during chemotherapy.	Resistome profiling

Table 1 provides a structured overview of pediatric ALL microbiome studies, facilitating transparent assessment of evidence quality and clear distinction between direct ALL findings and extrapolated data.

a Studies marked with an asterisk represent populations related to but not exclusively pediatric ALL (HSCT or general pediatric hematologic malignancies). Evidence from these studies is extrapolated to pediatric ALL with appropriate caution. 16S, 16S ribosomal RNA gene sequencing; shotgun, shotgun metagenomics; NR, not reported; HSCT, hematopoietic stem cell transplantation; SCFA, short-chain fatty acid; EN, enteral nutrition; PN, parenteral nutrition; aGVHD, acute graft-versus-host disease.

## 4 Mechanistic links between gut microbiota and leukemia

### 4.1 Immune modulation

Intestinal microbial communities function as the predominant external modulator of immunological maturation and ongoing immune competence, mediating their effects through elaborate bidirectional molecular communication with host immunocytes (61). During critical developmental windows in early childhood, symbiotic microorganisms actively participate in programming the nascent immune apparatus, instructing tolerance mechanisms directed against innocuous environmental antigens while simultaneously preserving effector capacity against genuine microbial threats (36). This immunological instruction encompasses the orchestrated differentiation and clonal expansion of CD4 ^+^ CD25 ^+^ FOXP3^+^ regulatory T lymphocytes, T-bet-expressing Th1 effector populations, ROR*γ*t-dependent Th17 subsets, and tissue-resident innate lymphoid cell families-each of which exhibits marked sensitivity to colonization with particular microbial species and their bioactive metabolic outputs (62).

Dysbiosis-defined as a pathological alteration in microbiota composition-may disrupt these immunoregulatory circuits in ways that could promote leukemogenesis. Preclinical and correlative evidence suggests that depletion of SCFA-producing bacteria is associated with reduced Treg differentiation and IL-10 production, potentially unleashing unchecked inflammatory responses that create a microenvironment conducive to malignant transformation. While SCFAs promote Treg differentiation in experimental models, whether this relationship is causal in human leukemia remains to be established (40). Conversely, expansion of pathobionts such as *Proteobacteria* and *Enterobacteriaceae are* associated with Toll-like receptor (TLR)-mediated signaling, with lipopolysaccharide (LPS) stimulation promoting the release of proinflammatory cytokines including tumor necrosis factor-alpha (TNF-alpha), interleukin-6 (IL-6), and IL-1beta that can enhance the proliferation and survival of pre-leukemic clones (63, 64).

Experimental evidence supports a causal role for microbiota-mediated immune modulation in leukemia development. Mechanistic insights from germ-free mice or animals treated with broad-spectrum antibiotics exhibit defective hematopoiesis, impaired neutrophil production, and altered responses to chemotherapeutic agents (56, 65). Emerging evidence from a single study suggests that specific bacterial consortia may restore granulopoiesis and enhance neutrophil recovery following chemotherapy-induced myelosuppression. Independent confirmation of this finding is needed before clinical relevance can be inferred (57). The bidirectional crosstalk between the gut microbiota and bone marrow hematopoietic activity, mediated in part by circulatory microbial metabolites and innate immune signaling, positions the microbiota as a modulator of both leukemia biology and treatment response.

### 4.2 Inflammation and leukemogenesis

Sustained inflammatory signaling forms a recognized enabling characteristic in multistep carcinogenesis, with the gastrointestinal microbiota representing a quantitatively important source of immunostimulatory molecular patterns (66). The compromised intestinal barrier concept-elaborated in Section 4.3-proposes that ecological perturbation within the gut lumen compromises epithelial tight junction integrity and mucosal defense mechanisms, facilitating the paracellular and transcellular passage of microbial components into portal and systemic circulation. Upon reaching extra-intestinal tissues, structural moieties including LPS, peptidoglycan fragments, and unmethylated CpG-rich bacterial DNA engage germline-encoded pattern recognition receptors expressed on macrophages, dendritic cells, and neutrophils, initiating canonical NF-*κ*B activation programs that sustain a chronically inflamed tissue microenvironment (63, 67).

In the context of childhood ALL, microbially driven inflammation may promote the second oncogenic “hit” required for leukemic transformation. Greaves and colleagues demonstrated that exposure of ETV6-RUNX1-transgenic mice to common pathogens induces chronic inflammatory responses that facilitate the acquisition of secondary genetic lesions in pre-leukemic B-cell progenitors (41, 44). Activation-induced cytidine deaminase (AID) and recombination-activating genes (RAG1/RAG2), enzymes normally responsible for immunoglobulin gene rearrangement, are aberrantly activated by inflammatory signaling and can induce off-target DNA damage in pre-leukemic cells-promoting clonal evolution toward overt leukemia (44).

Beyond direct genotoxic effects, microbial products can modulate the bone marrow microenvironment. Secondary bile acids-produced by microbial transformation of primary bile acids-have emerged as signaling molecules influencing T-cell differentiation and immune surveillance (49). The heightened bile acid-transforming capacity observed in treatment-naïve ALL patients may have immunomodulatory consequences that influence disease biology. Similarly, alterations in tryptophan metabolism along the kynurenine pathway-regulated by microbial enzymes and indoleamine 2,3-dioxygenase (IDO)-can suppress antitumor immunity and promote immune tolerance (68). These metabolic axes represent promising targets for mechanistic investigation and therapeutic intervention.

### 4.3 Intestinal barrier dysfunction

The intestinal epithelium forms the body's largest mucosal surface and serves as a critical barrier separating luminal contents from the underlying immune system. Tight junction proteins-including claudins, occludin, and zonula occludens-1 (ZO-1)-maintain barrier integrity by sealing the paracellular space between epithelial cells (69). Chemotherapy agents, particularly methotrexate and cytarabine, directly damage the intestinal epithelium, causing villous atrophy, crypt loss, and tight junction disruption-a condition clinically manifested as gastrointestinal mucositis (70, 71).

Dysbiosis amplifies barrier dysfunction through multiple mechanisms. Depletion of SCFA-producing bacteria deprives colonocytes of butyrate-their preferred energy substrate-resulting in epithelial atrophy and impaired tight junction expression (72). Expansion of mucolytic species such as *Ruminococcus gnavus* and *Ruminococcus torques* degrades the protective mucus layer, bringing luminal bacteria into closer proximity with the epithelial surface (20, 73). Pathobiont overgrowth introduces virulence factors and inflammatory stimuli that further compromise barrier integrity.

The consequences of barrier disruption extend far beyond the gut. Increased intestinal permeability enables bacterial translocation-the passage of viable bacteria or microbial products across the epithelium into mesenteric lymph nodes and systemic circulation (74). In pediatric ALL patients undergoing chemotherapy, microbial translocation is a major contributor to bloodstream infections and febrile neutropenia (75). The correlation between elevated plasma LPS-binding protein (LBP)-a marker of LPS translocation-and increased intestinal permeability (measured by lactulose: rhamnose ratio) has been validated in pediatric transplant recipients, highlighting the clinical relevance of the gut barrier as a determinant of infectious risk (76).

### 4.4 Microbial metabolites

Microbial metabolites constitute a critical interface through which the gut microbiota influences host physiology, with SCFAs representing the best-characterized class. Acetate, propionate, and butyrate are produced through anaerobic fermentation of dietary fibers and resistant starches by specific bacterial taxa: *Faecalibacterium prausnitzii*, *Roseburia* species, *Eubacterium rectale*, and select *Bacteroides* species are predominant butyrate and propionate producers, while acetate is generated by a broader range of taxa (40, 77).

SCFAs exert multifaceted effects relevant to leukemia biology. Butyrate functions as a potent HDAC inhibitor, modulating gene expression through increased histone acetylation at gene promoters and enhancers (78). At high concentrations, butyrate has been reported to induce apoptosis in human acute leukemia cell lines-including U937 monocytic leukemia cells-through caspase-3 activation and downregulation of pro-survival signaling (79). This finding, derived from a single *in vitro* study, requires independent confirmation before clinical relevance can be inferred. This direct anti-leukemic activity has prompted exploration of butyrate and its derivatives as therapeutic agents in hematological malignancies. Propionate and butyrate also function as unique epigenetic regulatory elements, serving as substrates for histone propionylation and butyrylation-post-translational modifications that link dietary intake, microbial metabolism, and gene expression programs (80).

Beyond epigenetic regulation, SCFAs signal through G-protein-coupled receptors GPR41 (FFAR3) and GPR43 (FFAR2) expressed on immune cells, enteroendocrine cells, and adipocytes (77). GPR-mediated signaling in colonic Tregs promotes anti-inflammatory IL-10 production and maintains mucosal tolerance (40). SCFAs also enhance intestinal barrier function by stimulating mucin production and tight junction protein expression (72). The profound depletion of SCFA-producing bacteria observed in ALL patients-both at diagnosis and during chemotherapy-thus has far-reaching consequences for immune homeostasis, epigenetic regulation, and barrier integrity.

Tryptophan metabolites represent another class of bioactive microbial products with relevance to leukemia. Microbial metabolism of tryptophan generates indole derivatives, indole-3-lactic acid (ILA), and kynurenine pathway metabolites that modulate aryl hydrocarbon receptor (AhR) signaling-a pathway critical for immune regulation and hematopoietic stem cell maintenance (68, 81). Recent studies have identified reduced microbial tryptophan metabolism as a feature of chemotherapy-induced dysbiosis, with potential implications for intestinal homeostasis and immune reconstitution (82). The enrichment of specific tryptophan-metabolizing taxa, such as *Lactobacillus reuteri* and *Clostridium sporogenes*, in healthy compared to ALL patients suggests that targeted restoration of these organisms might support AhR-dependent mucosal barrier maintenance and immune tolerance during treatment.

Bile acid metabolism is an additional metabolic axis linking microbiota function to leukemia biology. Primary bile acids synthesized in the liver are converted by gut bacteria into secondary bile acids, including deoxycholic acid (DCA) and lithocholic acid (LCA). This function as signaling molecules through the farnesoid X receptor (FXR) and the G-protein-coupled bile acid receptor 1 (TGR5) (49). These receptors are expressed on intestinal epithelial cells, immune cells, and hematopoietic progenitors, mediating effects on intestinal barrier function, inflammatory responses, and cellular differentiation. The elevated bile acid-transforming capacity observed in treatment-naïve ALL patients-particularly the enrichment of Clostridium scindens, a primary 7-alpha-dehydroxylating species-suggests that microbial bile acid metabolism may be dysregulated even before chemotherapy begins, with potential consequences for immune homeostasis and treatment response.

### 4.5 Infection susceptibility and hematologic recovery

The gut microbiota plays an important role in shaping infection risk during chemotherapy-induced neutropenia. Under normal conditions, the microbiota provides “colonization resistance"-ecological competition and antimicrobial substance production that prevents pathogen expansion (83). Chemotherapy disrupts this protective ecosystem, eliminating commensal anaerobes that suppress pathobiont growth while simultaneously creating ecological niches favorable to opportunistic organisms.

Emerging evidence suggests that that specific microbiota signatures show preliminary predictive value for infection risk and hematologic recovery in ALL. Andersen and colleagues found that baseline abundances of *Ruminococcaceae* and *Lachnospiraceae* genera predicted the risk of prolonged neutropenia following induction chemotherapy, with *Enterococcus* dominance associated with delayed neutrophil recovery (56). These observations align with mechanistic studies in germ-free mice demonstrating that certain gut bacteria facilitate neutrophil recovery after chemotherapy by stimulating granulopoiesis within the bone marrow and modulating chemokine signaling (CXCL1, CXCL8) that controls neutrophil recruitment (56, 57).

Conversely, *Enterococcaceae* and *Streptococcaceae* dominance during chemotherapy has been associated with subsequent febrile neutropenia and diarrheal illness (22). Hakim and colleagues demonstrated that a baseline microbiota characterized by *Proteobacteria* enrichment predicted febrile neutropenia during ALL therapy, while *Enterococcaceae* dominance (relative abundance ≥30%) predicted significantly greater risk of subsequent infectious complications (22). These predictive associations suggest that microbiota profiling could inform risk-stratified infection prevention strategies, potentially guiding prophylactic antibiotic selection, immunomodulatory interventions, or enhanced monitoring for high-risk patients. The development of clinically feasible microbiota-based risk scores-incorporating baseline microbiota composition, clinical variables, and treatment parameters-is an urgent priority for translational research in pediatric ALL (Figure 2).

**Figure 2:**
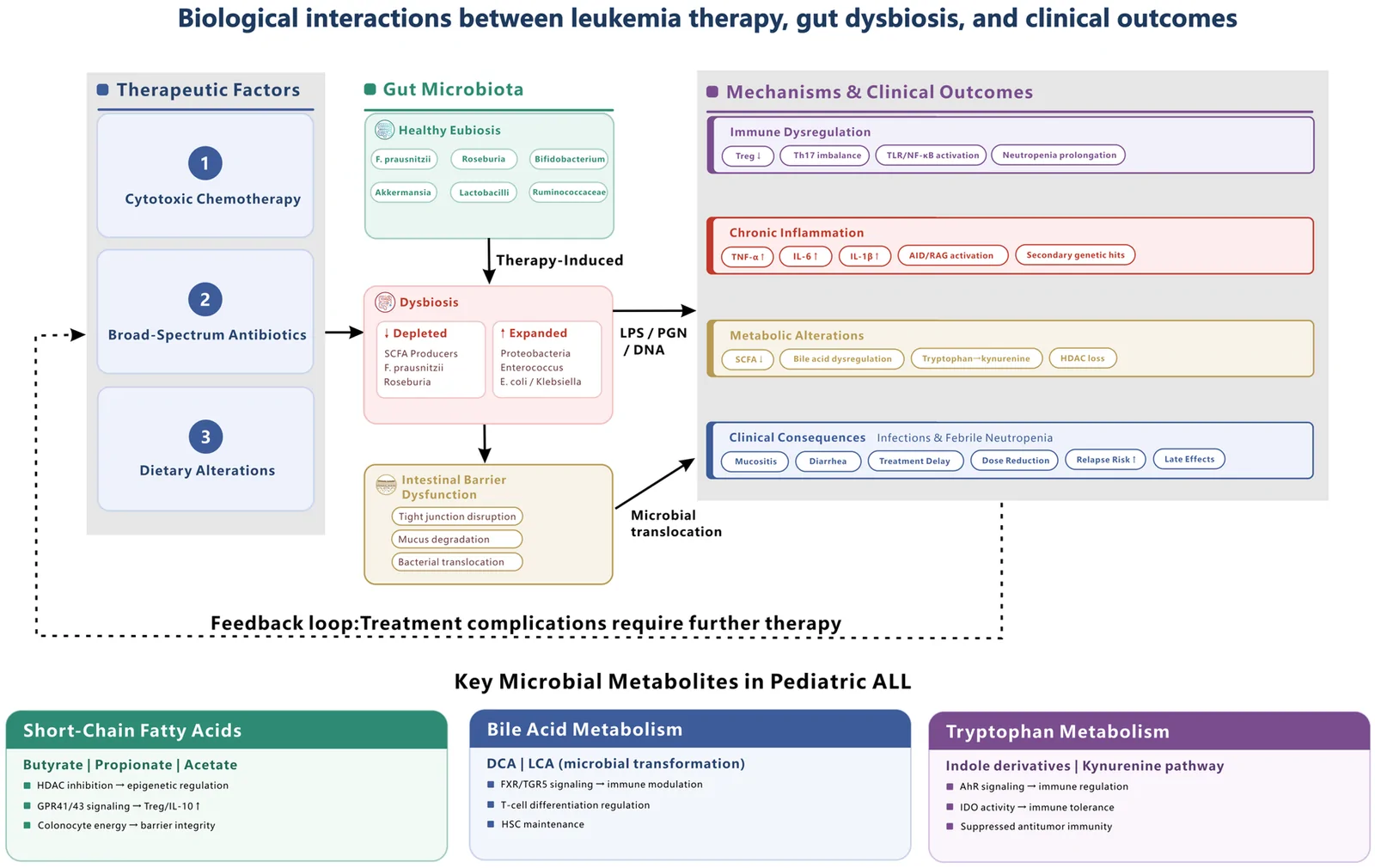
Gut microbiota-leukemia axis in pediatric ALL.

Cytotoxic chemotherapy, broad-spectrum antibiotics, and dietary alterations contribute the transition from healthy gut eubiosis to dysbiosis, marked by loss of short-chain fatty acid (SCFA)–producing commensals (e.g., *Faecalibacterium prausnitzii*, *Roseburia*, *Bifidobacterium*) and expansion of pathobionts (e.g., *Proteobacteria*, *Enterococcus*, *Enterobacteriaceae*). Dysbiosis disrupts intestinal barrier integrity, promoting microbial translocation of lipopolysaccharide (LPS), peptidoglycan, and bacterial DNA into systemic circulation. These products activate three convergent pathways: (1) immune dysregulation (Treg depletion, Th17 imbalance, TLR/NF-*κ*B activation); (2) chronic inflammation (TNF-α/IL-6/IL-1β elevation, AID/RAG activation, secondary genetic hits); and (3) metabolic alterations (SCFA depletion, bile acid dysregulation, tryptophan shunting to kynurenine, and loss of HDAC inhibition). Collectively, these mechanisms produce infectious complications, gastrointestinal toxicities, treatment delays, and increased relapse risk-establishing a feedback loop that perpetuates dysbiosis. The lower panel summarizes three major microbial metabolite classes and their relevant signaling pathways: SCFAs (GPR41/43, HDAC inhibition), secondary bile acids (DCA/LCA; FXR/TGR5), and tryptophan metabolites (AhR/IDO).

## 5 Gut microbiota and clinical outcomes in pediatric leukemia

### 5.1 Infection and febrile neutropenia

Neutropenic fever is the most common and serious adverse event of cytotoxic therapy for childhood ALL. Incidence reaches 60% during initial induction, requiring inpatient care, empirical broad-spectrum antibiotics, and chemotherapy schedule modifications (22, 84). The gut microbiota serves as both a source of infectious organisms and a determinant of infection risk. For the mechanistic basis of microbiota-mediated infection susceptibility, see Section 4.5; for antibiotic-specific effects on infection risk, see Section 6.2. Here, we focus on the clinical epidemiology and predictive value of microbiota composition.

In a cohort of 199 children with ALL, Hakim and colleagues documented 1,313 microbiologically derived infections during therapy, with gut-derived organisms representing a substantial proportion (22). Baseline gut microbiota composition was highly predictive: patients harboring microbiota enriched in Proteobacteria before chemotherapy initiation were significantly more likely to develop febrile neutropenia during subsequent treatment phases (22). Enterococcaceae dominance (relative abundance ≥30%) conferred significantly elevated risk of febrile neutropenia (OR 2.8, 95% CI 1.4–5.6, *p* = 0.003), while Streptococcaceae dominance predicted diarrheal illness (22). Wang and colleagues further demonstrated that baseline Bifidobacterium longum abundance predicted infectious complications during induction chemotherapy (AUC = 0.720) (23).”

“The predominant causative organisms span Gram-positive cocci (coagulase-negative staphylococci, *Staphylococcus aureus*, Enterococcal strains), *Enterobacteriaceae* and non-fermenting Gram-negative rods (*E. coli*, *Klebsiella pneumoniae*, *Pseudomonas aeruginosa*), and opportunistic fungal organisms (Candida species) (6, 85).

Beyond compositional markers, functional microbiome features-including reduced fecal SCFA concentrations and impaired tryptophan metabolism-have been associated with increased infection susceptibility in hematological malignancies (for mechanistic discussion, see Sections 4.4 and 4.5) (55, 82). Machine learning approaches incorporating multi-dimensional microbiota data have shown preliminary promise in developing predictive models for bloodstream infections (see Section 8.3) (86, 87).”

### 5.2 Treatment toxicity

Chemotherapy-related gastrointestinal injury ranks among the most ubiquitous adverse consequences of ALL treatment, with clinically evident mucositis developing in approximately 40% of children and contributing substantially to symptom burden, analgesic requirements, and compromised nutritional status (88). The molecular pathogenesis of mucotoxicity encompasses direct antiproliferative injury to rapidly cycling gastrointestinal epithelium, oxidative stress-mediated cellular damage through free radical generation, and feed-forward amplification of inflammatory injury via cytokine networks (TNF-α, NF-*κ*B-dependent transcriptional programs) (70, 89). The resident microbiota critically modulates this injury paradigm: ecological disruption erodes mucosal resilience, while microbial-derived inflammatory agonists potentiate the tissue damage response.

The Firmicutes-to-Bacteroidetes ratio has been proposed as an indicator of dysregulated microbiota in mucositis pathogenesis (18). Depletion of butyrate-producing bacteria impairs epithelial energy metabolism and barrier function, while expansion of pathobionts introduces inflammatory stimuli that amplify mucosal damage. In a cohort of 39 ALL-diagnosed children and 39 healthy controls, only ALL patients developed oral mucositis (15.4%) and candidiasis (2.6%), with *Lactobacillales* enrichment observed in affected patients (90). Probiotic strains including *Lactobacillus rhamnosus* have demonstrated protective effects in preclinical models, attenuating proinflammatory cytokine levels (IL-1beta, IL-6, TNF-alpha) and upregulating tight junction proteins (claudin, occludin, ZO-1) and mucin-2 production (91).

Diarrheal illness complicates approximately 37% of ALL treatment courses and is associated with dehydration, electrolyte disturbances, and need for treatment modification (22). Chemotherapy-induced dysbiosis-particularly *Streptococcaceae* dominance-has been associated with diarrheal episodes, likely reflecting impaired carbohydrate fermentation, altered bile acid metabolism, and direct pathobiont-mediated secretory mechanisms (18, 22, 50). Nutritional interventions that promote microbial stability and SCFA production may offer complementary approaches to conventional antidiarrheal therapy.

### 5.3 Treatment efficacy

The emerging field of pharmacomicrobiomics examines how the gut microbiota influences drug metabolism, bioavailability, and therapeutic efficacy (92). In adult oncology, specific bacterial taxa have been shown to metabolize chemotherapeutic agents (92), modulate drug-induced immunogenic cell death, and are associated with treatment response (93). While analogous studies in pediatric ALL remain limited, several lines of evidence suggest that microbiota composition may influence chemotherapy outcomes. Evidence for microbiota-mediated effects on chemotherapy efficacy in pediatric ALL remains speculative and extrapolated from adult oncology studies. Direct pediatric evidence is lacking, and the clinical significance of microbiota-drug interactions for ALL outcomes requires dedicated investigation.

Methotrexate-a cornerstone of ALL therapy-undergoes bacterial metabolism in the intestine that may affect systemic exposure. Preclinical evidence from animal models demonstrate that methotrexate administration alters gut microbiota composition while microbiota status reciprocally influences drug pharmacokinetics (93, 94). Dexamethasone and prednisone, corticosteroids used throughout ALL therapy, also exhibit bidirectional interactions with the microbiota: these agents directly modulate microbial composition while gut bacteria metabolize steroids and generate anti-inflammatory signals that may influence treatment response (18). The clinical significance of these interactions for ALL outcomes requires further investigation but is a compelling target for translational research.

### 5.4 Prognosis and relapse

The relationship between gut microbiota and leukemia prognosis is a frontier area with profound clinical implications. Emerging evidence from adult hematological malignancies suggests that microbiota composition at diagnosis and during therapy correlates with treatment response, relapse risk, and overall survival (95). In acute myeloid leukemia (AML), higher baseline microbiota diversity has been associated with improved relapse-free survival, while *Enterobacteriaceae* dominance predicts worse outcomes (95).

Direct evidence linking microbiota composition to ALL relapse risk remains sparse but is accumulating. The microbiota's influence on treatment tolerance-through modulation of infectious complications, mucositis severity, and dose intensity-may indirectly affect leukemia control. Patients experiencing treatment delays or dose reductions due to infections or toxicity may have inferior disease-free survival (96). Microbiota-mediated immune modulation could influence the graft-versus-leukemia effect in patients undergoing hematopoietic stem cell transplantation for high-risk or relapsed disease. As multi-omic profiling becomes integrated into pediatric ALL trials, the prognostic value of microbiota features will likely become clearer.

Direct evidence linking gut microbiota composition to ALL relapse risk remains limited. While baseline dysbiosis has been associated with infectious complications during therapy (23), no prospective study has yet demonstrated that pretreatment microbiota profiles predict leukemic relapse independently of conventional risk factors (e.g., cytogenetics, minimal residual disease). In long-term survivors, persistent depletion of butyrate-producing taxa correlates with markers of chronic systemic inflammation and T-cell activation (53), raising the possibility that treatment-induced dysbiosis contributes to the elevated risk of metabolic syndrome, cardiovascular disease, and secondary immune dysfunction observed in this population. Whether microbiota-targeted interventions during or after therapy can modify these long-term outcomes-or reduce relapse risk-represents a critical unanswered question that warrants dedicated prospective investigation.

## 6 Impact of leukemia therapy on gut microbiota

### 6.1 Chemotherapy-induced dysbiosis

Cytotoxic chemotherapy profoundly alters the intestinal microbiota, as detailed in Section 3.2. Rather than repeating these taxonomic findings, we focus here on the clinical implications. The loss of colonization resistance-conferred by diverse anaerobic commensals-creates ecological niches favoring facultative and aerobic opportunists, particularly Enterococcaceae, Streptococcaceae, and Enterobacteriaceae (83). This ecological shift directly increases the risk of bloodstream infections and febrile neutropenia, which represent the leading causes of treatment-related morbidity in pediatric ALL (22). Concurrently, depletion of SCFA-producing bacteria impairs epithelial energy metabolism and barrier integrity, predisposing to mucositis and microbial translocation (20, 72). The magnitude of dysbiosis varies by chemotherapy intensity and concurrent antibiotic exposure, creating individualized risk profiles that warrant consideration in supportive care planning.

### 6.2 Antibiotics and antifungal agents

Broad-spectrum antibiotics are administered to approximately 60% of children during initial induction for febrile neutropenia or empirical infection coverage (22, 84). Antibiotic-driven depletion of anaerobic commensals creates ecological vacuums, with the magnitude and spectrum of disruption varying by antibiotic class. Fluoroquinolones, which exert broad anaerobic coverage, induce more profound dysbiosis than beta-lactams, which primarily target aerobic Gram-negative organisms (83, 97). The clinical consequence is a shift from diverse, anaerobe-dominant communities to simplified ecosystems dominated by facultative pathogens, which has been associated with subsequent infectious complications (see Section 5.1 for predictive markers and clinical outcomes).

### 6.3 Diet during treatment

Nutritional status deteriorates significantly during ALL therapy due to chemotherapy-induced anorexia, nausea, vomiting, mucositis-related oral pain, and altered taste perception (98). Reduced oral intake, combined with the metabolic demands of cancer and treatment, frequently results in weight loss, protein-energy malnutrition, and micronutrient deficiencies. These nutritional changes have direct consequences for the gut microbiota: dietary fiber intake-the primary substrate for SCFA-producing bacteria-declines dramatically when patients consume restricted, palatable diets low in plant foods (99).

The shift from enteral to parenteral nutrition in patients unable to tolerate oral feeding further compounds microbiota disruption. Studies in hematopoietic stem cell transplant recipients demonstrate that total parenteral nutrition (TPN) is associated with gut mucosal atrophy, loss of microbial diversity, and expansion of pathogenic taxa, whereas enteral nutrition (EN) preserves microbial community structure and SCFA production (58, 100). TPN provides complete nutritional support intravenously and is associated with marked gut microbiota disruption due to intestinal atrophy and lack of luminal substrate. Partial parenteral nutrition (PPN), which supplements rather than replaces enteral intake, may preserve some microbial diversity but still alters community composition compared to full enteral feeding. In pediatric ALL, where prolonged periods of poor oral intake are common, nutritional support strategies that maintain enteral feeding and provide adequate fiber may help preserve microbiota integrity-a rationale explored further in Section 7.

Treatment-induced anorexia represents one of the most pervasive nutritional challenges in pediatric ALL therapy, as cytotoxic agents-particularly corticosteroids, vincristine, and asparaginase-directly suppress appetite through central mechanisms involving hypothalamic neuropeptide signaling and alter taste perception, leading to profound dietary intake reduction that often precedes visible weight loss (8). Although modern antiemetic protocols partially mitigate chemotherapy-induced nausea and vomiting, these symptoms still compromise voluntary food intake during the most intensive treatment phases. Concurrently, oral mucositis, which develops in approximately 40% of children receiving cytotoxic therapy, causes significant pain with chewing and swallowing, leading to selective avoidance of textured, acidic, or temperature-variable foods (88). Nausea conditioned by prior emetic episodes can additionally produce anticipatory aversions to previously tolerated foods, progressively narrowing the acceptable dietary repertoire and increasing reliance on energy-dense, nutrient-poor comfort foods. When oral intake becomes insufficient to meet metabolic demands, enteral nutrition (EN) via nasogastric or gastrostomy tube represents the preferred alternative, as it preserves gut mucosal integrity and microbiota diversity compared with TPN; however, TPN may be unavoidable during severe mucositis, ileus, or prolonged agranulocytosis, and PPN, which supplements rather than replaces enteral intake, offers an intermediate strategy that partially maintains luminal substrate delivery (101).

The source and preparation of food during treatment also warrant careful attention: hospital-provided meals, while nutritionally formulated to meet macronutrient and micronutrient requirements, may lack the variety and palatability of home-prepared foods, whereas transitioning to home-based nutrition during outpatient maintenance phases can improve dietary diversity and patient autonomy, though family education regarding food safety-particularly the strict avoidance of unpasteurized dairy products, undercooked meats, and raw foods in neutropenic patients-is essential to prevent foodborne infections. Finally, systematic nutritional monitoring, including serial anthropometric assessment such as weight-for-height and mid-upper arm circumference, serum prealbumin and vitamin D measurement, and prospective dietary intake recording, should be integrated into routine clinical care; early identification of nutritional deterioration enables timely intervention and may mitigate both treatment-related toxicity and microbiota perturbation, thereby supporting optimal treatment outcomes.

## 7 Nutritional and microbiota-targeted interventions

### 7.1 Probiotics

Live microbial preparations administered in sufficient quantities to elicit favorable physiological responses-commonly termed probiotics-have garnered the most substantial research attention among microbiota-directed interventions within oncology (102). Species belonging to the genera *Lactobacillus* and *Bifidobacterium* have accumulated evidence supporting their utility in preventing antimicrobial-associated diarrheal illness, fine-tuning mucosal and systemic immune reactivity, and reinforcing gastrointestinal epithelial barrier integrity across diverse patient populations (103). Within the cancer setting, probiotic supplementation has demonstrated preliminary efficacy in ameliorating chemotherapy-related oral and intestinal mucositis, reducing stool frequency during treatment, and potentially lowering infection incidence, though robust pediatric ALL-specific clinical trial data remain comparatively scarce (104).

The safety of probiotic administration in immunocompromised cancer patients warrants careful consideration. Systematic reviews have generally found probiotics to be well tolerated in oncology populations, with no significant increase in serious adverse events or bacteremia in randomized controlled trials (105). Yet, case reports of probiotic-related infections-including *Lactobacillus* bacteremia and *Bifidobacterium* bacteremia-have occurred in profoundly immunocompromised patients with central venous catheters, underscoring the need for strain-specific safety assessment and careful patient selection (105, 106).

In pediatric oncology, small pilot studies have explored probiotic supplementation during chemotherapy. A pilot trial using *Enterococcus faecium* M-74 in pediatric oncology patients was conducted in 2006 (107). This study has not been replicated in the subsequent two decades, and the use of *Enterococcus* species in immunocompromised patients raises safety concerns given documented cases of enterococcal bacteremia in this population. Probiotic administration was not associated with increased incidence of infectious complications in pediatric patients receiving chemotherapy across several small trials (99). Yet, these studies were limited by small sample sizes, heterogeneous populations, and short follow-up durations.

The strain-specificity of probiotic effects demands attention. Different *Lactobacillus* and *Bifidobacterium* strains exhibit distinct immunomodulatory capacities, colonization efficiencies, and metabolic activities (108). *Lactobacillus rhamnosus* GG has demonstrated efficacy in preventing antibiotic-associated diarrhea, while *Bifidobacterium longum* subsp. *infantis* excels at metabolizing human milk oligosaccharides and promoting barrier function (103). For pediatric ALL, strains with documented capacity to stimulate SCFA production, enhance tight junction expression, and suppress pathobiont expansion may offer the greatest therapeutic potential. Future trials should prioritize well-characterized strains with mechanisms of action relevant to the specific pathophysiology of chemotherapy-induced dysbiosis.

Safety Considerations and Clinical Guidelines. Despite promising findings from individual trials, probiotic use in severely immunocompromised patients remains controversial. The European Society for Clinical Nutrition and Metabolism (ESPEN) guidelines (109) and the American Society of Clinical Oncology (ASCO) recommend against routine probiotic administration in severely neutropenic patients (absolute neutrophil count <500/*μ*L) due to insufficient safety data and documented cases of probiotic-associated bacteremia (109, 110). In pediatric ALL, where prolonged neutropenia is common during induction, these safety concerns are particularly relevant. We emphasize that probiotic interventions in this population should be confined to well-designed clinical trials with rigorous safety monitoring and institutional review board approval.

### 7.2 Prebiotics and dietary fiber

Substrates that resist host enzymatic hydrolysis in the upper gastrointestinal tract and undergo selective fermentation by desirable colonic bacteria-classified collectively as prebiotics-represent another therapeutic approach to microbiota modulation (111). Well-characterized prebiotic compounds include short-chain fructooligosaccharides (scFOS), galactooligosaccharides (GOS) derived from lactose, inulin-type fructans, and granular resistant starch varieties, each of which preferentially supports proliferation and metabolic activity of butyrate and other SCFA-producing populations including bifidobacterial and lactobacillary species (112). Controlled feeding studies in healthy infant cohorts have demonstrated that GOS-fortified formula promotes enrichment of *Bifidobacterium* and *Lactobacillus* populations with concurrent suppression of *Clostridium* lineages, effects accompanied by measurable elevations in fecal SCFA output (113).

The rationale for prebiotic supplementation in pediatric ALL is compelling. Chemotherapy-induced dysbiosis is characterized by depletion of SCFA producers, and prebiotics provide the substrate necessary to support their recovery. Resistant starch-found in cooked and cooled potatoes, rice, and legumes-has shown particular promise: dietary supplementation with resistant potato starch significantly elevated fecal butyrate levels in a clinical study, offering a practical nutritional route to bolster SCFA-mediated immune modulation (114). High-fiber diets enrich SCFA producers such as Bacteroides acidifaciens and improve intestinal epithelial regeneration following radiation injury (115).

In the context of pediatric cancer, prebiotic supplementation must navigate the practical constraints of treatment: reduced oral intake, mucositis-related pain, and taste alterations may limit tolerance of fiber-rich foods or supplements. Nevertheless, creative delivery strategies-such as incorporating prebiotics into palatable enteral formulas or beverages-may enhance feasibility. The safety profile of prebiotics is favorable, with the primary adverse effects being mild gastrointestinal symptoms (bloating, flatulence) that are generally self-limiting (111). Unlike probiotics, prebiotics do not introduce live organisms and thus carry no risk of translocation or infection in immunocompromised hosts-a significant safety advantage in pediatric ALL. However, their fermentation can produce gas and bloating that may exacerbate gastrointestinal symptoms in patients with mucositis. Moreover, in severely dysbiotic ecosystems, prebiotics may inadvertently stimulate pathobiont growth rather than beneficial commensals, highlighting the importance of restoring ecological stability before prebiotic supplementation.

### 7.3 Synbiotics and postbiotics

Synbiotics combine probiotics with prebiotics in a single formulation, theoretically enhancing the survival and colonization of beneficial bacteria while simultaneously providing their preferred metabolic substrates (116). In pediatric populations, synbiotic supplementation (GOS combined with *Lactobacillus fermentum* or *Bifidobacterium breve*) has increased *Bifidobacterium* abundance and reduced pathogen colonization in clinical trials involving healthy infants (117). The application of synbiotics to pediatric oncology is nascent but conceptually attractive: delivering both beneficial organisms and their growth substrates may achieve more robust and durable microbiota modification than either intervention alone.

Postbiotics-non-viable bacterial products or metabolites that confer health benefits-represent an emerging concept with particular relevance to immunocompromised patients (118). SCFAs (butyrate, propionate, acetate), bacterial cell wall components, exopolysaccharides, and biosurfactants constitute major classes of postbiotics with documented immunomodulatory, barrier-enhancing, and antimicrobial properties. The advantage of postbiotics over live probiotics lies in their complete safety profile: without viable organisms, there is no risk of infection, translocation, or antibiotic resistance gene transfer in neutropenic patients (118).

Butyrate has emerged as a particularly promising postbiotic in cancer care. The biphasic model of butyrate action-whereby low concentrations promote epithelial proliferation and high concentrations induce apoptosis- remains a working hypothesis that has been contested in subsequent studies. The concentration thresholds defining these divergent effects have not been established *in vivo*, and the clinical relevance of this model in pediatric ALL remains speculative (119). This context dependency suggests that the timing, dose, and route of butyrate supplementation must be carefully optimized for therapeutic benefit in pediatric ALL.

### 7.4 Dietary interventions

Enteral nutrition (EN)-the delivery of nutritionally complete formulas directly into the gastrointestinal tract via nasogastric or nasojejunal tubes-offers a practical approach to maintaining gut mucosal integrity and supporting microbiota homeostasis during periods of poor oral intake (100). A landmark study in pediatric hematopoietic stem cell transplant recipients demonstrated that EN promoted faster recovery of gut microbiota diversity and SCFA production compared to parenteral nutrition (PN) in adult hematopoietic stem cell transplant patients (100). Whether comparable benefits occur in pediatric ALL patients-and the mechanistic basis for any such effect-remains to be established. EN-fed patients exhibited stable *Lachnospiraceae* abundance, increased *Blautia* and *Dorea* (SCFA producers), and higher fecal concentrations of butyrate, acetate, and propionate post-transplant. none of the EN patients developed bloodstream infections, whereas seven PN-treated patients developed eleven documented bloodstream infections attributed to *Staphylococcus*, *Enterococcus*, and *Streptococcus* (100).

A meta-analysis comparing EN and PN in hematopoietic stem cell transplantation showed that EN reduced the incidence of acute graft-versus-host disease (aGVHD), particularly severe grade III-IV aGVHD and gut aGVHD, potentially due to improved gut eubiosis in enterally fed patients (120). These findings have direct implications for pediatric ALL patients, who frequently require nutritional support during intensive treatment phases. Yet, practical challenges-including poor tolerance of nasogastric tubes, nausea, and gastrointestinal symptoms-have limited the widespread adoption of EN in this population (121).

Microbiota-supportive dietary patterns extend beyond enteral feeding to encompass the overall quality of dietary intake. The Mediterranean diet-rich in fiber, polyphenols, and unsaturated fats-has been associated with increased fecal SCFA levels, enrichment of Prevotella and fiber-degrading Firmicutes, and favorable microbial metabolic profiles in clinical trials in healthy adults (45). Evidence for this dietary pattern in pediatric cancer patients is currently lacking, and any recommendation would be extrapolative. While pediatric ALL patients may be unable to adhere to complex dietary patterns during acute treatment, the principles of microbiota-supportive nutrition-adequate fiber, minimization of ultra-processed foods, inclusion of fermented foods-can guide nutritional counseling. As treatment advances and oral intake improves, structured dietary interventions may help restore microbial diversity and metabolic function.

Polyphenols-including flavonoids, phenolic acids, and tannins-are metabolized by gut bacteria into bioactive compounds such as urolithins and equol, which exhibit anti-inflammatory and antioxidant properties. These microbial metabolites may modulate NF-*κ*B signaling and enhance intestinal barrier function. However, polyphenol bioavailability is highly variable, depending on baseline microbiota composition and dietary matrix. Clinical evidence for polyphenol supplementation in pediatric ALL is currently limited to preclinical models and small observational studies, and this area remains an emerging field requiring dedicated investigation. Polyphenols-plant-derived compounds abundant in fruits, vegetables, tea, and cocoa-represent another dietary component with microbiota-modulating properties. These compounds are poorly absorbed in the small intestine and reach the colon, where they are metabolized by gut bacteria into bioactive derivatives with antioxidant, anti-inflammatory, and antimicrobial properties (122). Flavonoids, in particularly, have been shown to promote the growth of beneficial taxa including *Bifidobacterium* and *Lactobacillus* while inhibiting pathogen expansion. The incorporation of polyphenol-rich foods into the diets of pediatric ALL patients-through berries, citrus fruits, green tea, and dark chocolate-may offer a palatable approach to supporting microbial health during and after treatment.

Fermented foods provide live microorganisms and microbial metabolites that may support gut ecosystem resilience. However, for immunocompromised patients-including those undergoing ALL induction-only pasteurized or heat-treated fermented foods should be considered, as unpasteurized products carry a risk of introducing opportunistic pathogens (e.g., *Listeria*, atypical mycobacteria). This safety distinction is critical and should be explicitly communicated to patients and families. Traditional fermented foods including yogurt, kefir, sauerkraut, and kimchi contain diverse bacterial consortia that can transiently colonize the gut and contribute to metabolic function. While direct evidence for fermented food consumption in pediatric ALL is lacking, the general safety of these foods and their potential to deliver beneficial organisms and bioactive metabolites makes them an attractive adjunct to conventional nutritional support. Care must be taken to ensure that fermented foods are pasteurized or otherwise microbiologically safe for immunocompromised patients, as unpasteurized products may contain opportunistic pathogens.

### 7.5 Fecal microbiota transplantation (FMT)

Fecal microbiota transplantation (FMT)-the transfer of fecal material from a healthy donor to a recipient-is the most potent microbiota-targeted intervention available, capable of durably reshaping the entire gut ecosystem (123). In adult oncology, FMT has shown remarkable efficacy in treating steroid-refractory graft-versus-host disease following allogeneic stem cell transplantation, achieving complete response rates of 38%–70% in prospective studies (124, 125). The mechanisms underlying FMT efficacy include restoration of microbial diversity, expansion of SCFA-producing bacteria, modulation of Treg populations, and suppression of inflammatory signaling (124).

Evidence for FMT in pediatric leukemia specifically is extremely limited, given the significant safety concerns in immunocompromised hosts. Yet, a recent systematic review of FMT in children confirmed that the procedure is generally well tolerated, with common adverse events including transient abdominal pain, diarrhea, and low-grade fever (126). However, this review included only 9 pediatric studies with substantial heterogeneity in donor selection, dosing, and clinical endpoints. The evidence base for FMT in pediatric oncology remains extremely limited, and safety data-particularly regarding infection risk in neutropenic patients-are insufficient to support clinical implementation outside of clinical trials. Serious adverse events-including rectal bleeding, aspiration pneumonia, and one death from sepsis in an immunocompromised patient-have been reported but are rare (0.7% attributable to FMT) (126). In adult hematology patients, FMT was found to be safe but did not significantly decrease infections in one randomized phase II trial (127), highlighting the need for further efficacy studies.

For pediatric ALL, FMT currently remains investigational and should be restricted to clinical trials or compassionate use in refractory cases-such as severe, recurrent *Clostridioides difficile* infection complicating chemotherapy. Critical considerations for pediatric FMT include donor selection (stringent screening for transmissible pathogens), route of administration (oral capsules, nasogastric tube, enema, or colonoscopy), dosing, and frequency of infusions (128). The long-term safety of FMT in children-including potential metabolic, immunological, and neuropsychiatric effects of altered microbial colonization-remains incompletely understood and requires longitudinal follow-up (126).

An alternative approach gaining traction is the use of defined bacterial consortia or “next-generation probiotics"-precisely characterized mixtures of beneficial strains that can be manufactured to pharmaceutical standards (129). These preparations offer the ecological restoration potential of FMT without the risks of uncharacterized donor material, representing a promising direction for future pediatric ALL trials (Figure 3).

**Figure 3:**
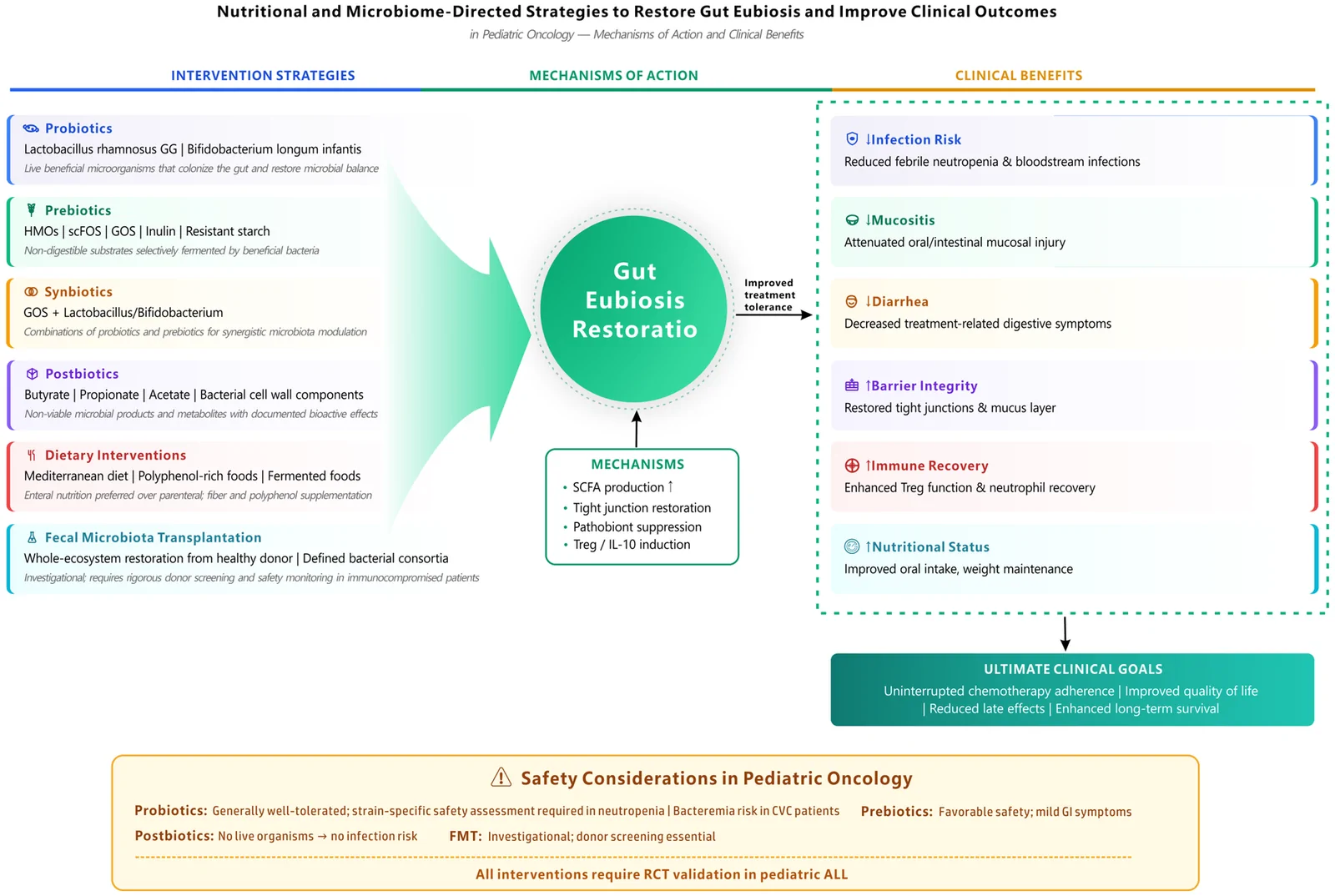
Microbiota-targeted interventions in pediatric ALL.

Six microbiota-directed strategies-probiotics, prebiotics, synbiotics, postbiotics, dietary interventions, and fecal microbiota transplantation (FMT)-converge on gut eubiosis restoration through shared mechanisms: enhanced SCFA production, tight junction restoration, pathobiont suppression, and Treg/IL-10 induction. These mechanisms translate into measurable clinical benefits, including reduced febrile neutropenia and bloodstream infections, attenuated mucositis, decreased diarrhea, restored intestinal barrier integrity, enhanced immune recovery, and improved nutritional status. Collectively, these outcomes support uninterrupted chemotherapy adherence and enhanced long-term survival. Probiotics require strain-specific safety monitoring during neutropenia; prebiotics and postbiotics carry favorable safety profiles; FMT remains investigational and requires rigorous donor screening. All interventions require prospective randomized controlled trial (RCT) validation in pediatric ALL before clinical adoption.

## 8 Methodological advances in microbiome research

### 8.1 Sequencing technologies

Selection of appropriate molecular profiling platforms fundamentally constrains both the breadth and granularity of microbiota-derived insights in pediatric ALL investigations. Amplification and sequencing of conserved hypervariable regions within the prokaryotic 16S ribosomal RNA gene has historically served as the predominant methodological approach, having been employed in foundational studies by Hakim, Rajagopala, and other research groups (20, 22). Practical attractions of amplicon-based 16S profiling encompass reduced per-sample analytical cost, comparatively streamlined computational processing requirements, and well-curated taxonomic reference frameworks (SILVA, Greengenes), factors that facilitate implementation in resource-constrained settings or investigations requiring substantial sample throughput (130). Short-read 16S rRNA gene sequencing has limited resolution for discriminating closely related strain variants or subspecies. Full-length 16S sequencing using PacBio or Oxford Nanopore platforms can achieve improved species-level resolution in some cases, though these approaches remain less widely adopted in clinical microbiota studies.

Shotgun metagenomic sequencing-involving the unbiased sequencing of all microbial DNA in a sample-has emerged as the gold standard for comprehensive microbiome characterization (131). However, it cannot distinguish viable from dead organisms, does not capture the active transcriptome, and remains more expensive and computationally demanding than 16S sequencing. Complementary approaches such as metatranscriptomics and culture-based methods are needed for a comprehensive understanding of microbiome activity. As demonstrated by Wang and colleagues in their 2024 study of ALL patients, shotgun metagenomics enables strain-level taxonomic resolution, functional gene profiling, and metabolic pathway reconstruction (23). This approach revealed functional alterations-including changes in carbohydrate metabolism, sulfur metabolism, and antibiotic resistance gene profiles-that remained invisible to 16S sequencing alone (23, 49). A recent comparative analysis of 16S and shotgun sequencing in colorectal cancer demonstrated that shotgun detects approximately nine times more species, exhibits lower sparsity, and provides greater alpha diversity estimates-though both methods can identify microbial signatures associated with disease when appropriately analyzed (132). Whether this resolution advantage is maintained in pediatric ALL-where antibiotic exposure and chemotherapy may further alter community composition-requires direct comparison.

For pediatric ALL research, the choice between technologies depends on study objectives. Descriptive epidemiological studies aiming to characterize broad compositional differences may be adequately served by 16S sequencing. Mechanistic investigations requiring functional insights, strain-level tracking, or antibiotic resistance gene profiling will benefit from shotgun metagenomics. The decreasing cost of shotgun sequencing-coupled with improved computational pipelines-suggests that this approach will become increasingly dominant in future pediatric ALL microbiota studies.

### 8.2 Multi-omics integration

The inherent complexity of host-microbial bidirectional communication in hematological malignancies defies capture by any individual analytical modality. Layered integration across multiple molecular profiling platforms-juxtaposing whole-community DNA sequencing (metagenomics) with comprehensive small-molecule profiling (untargeted metabolomics), genome-wide transcript abundance measurements (transcriptomics), protein-level quantification (proteomics), and chromatin modification mapping (epigenomics)-provides a more complete conceptual scaffold for interrogating these dynamic biological systems (133). Within the specific context of pediatric ALL, paired shotgun metagenomic and fecal metabolomic characterization has verified that numerical depletion of fiber-fermenting bacterial populations corresponds to quantifiable diminution in stool concentrations of butyrate, propionate, and acetate (49, 55). Parallel interrogation of host transcriptional programs-whether in mucosal tissue specimens or circulating immune cell fractions-alongside microbiota composition data illuminates the mechanistic pathways through which microbial cues influence immunological and inflammatory states.

Metabolomics deserves particular emphasis as a complementary layer to metagenomic profiling. While metagenomics predicts metabolic capacity, metabolomics measures the actual metabolites present in biological samples-bridging the gap between microbial gene content and functional output (134). Targeted metabolomic panels quantifying SCFAs, bile acids, tryptophan metabolites, and microbially modified host compounds can provide clinically actionable biomarkers for infection risk, treatment toxicity, and nutritional status (55, 82). The integration of metabolomic and metagenomic datasets through computational correlation networks enables the identification of specific microbe-metabolite relationships that contribute host phenotypes.

### 8.3 Machine learning approaches

The high dimensionality, compositional nature, and inter-individual variability of microbiota data pose analytical challenges that have spurred the adoption of machine learning (ML) methods (135). Supervised ML algorithms-including random forests, support vector machines, and neural networks-can identify microbial signatures predictive of clinical outcomes, often capturing complex, non-linear relationships invisible to conventional statistical tests. Unlike traditional hypothesis-driven analyses that test pre-specified associations, ML approaches enable data-driven discovery of predictive features across the entire microbiota landscape, potentially revealing unexpected microbial predictors that would be overlooked by conventional methods. The application of ML to microbiota data requires careful attention to cross-validation strategies, feature selection methods, and model interpretability to ensure robust and biologically meaningful findings.

In the context of febrile neutropenia and infection prediction, ML models have shown considerable promise. Gallardo-Pizarro and colleagues developed a machine learning model using the KAMILA clustering algorithm to stratify hematological malignancy patients with febrile neutropenia into distinct risk clusters for bloodstream infection (87). Three clusters were identified: a low-risk cluster with 3.9% bloodstream infection rate, an intermediate-risk cluster with 26.7% infection rate, and a high-risk cluster with 37.6% infection rate. High-risk cluster features included acute leukemia diagnosis, cyclophosphamide exposure, and prolonged hospitalization (87). In pediatric populations, Alali and colleagues (2022) developed a machine learning model for predicting bloodstream infections in febrile neutropenia in a general pediatric cancer population (not specifically ALL). This model has not been externally validated, and its generalizability to pediatric ALL-where chemotherapy regimens and infection profiles differ-remains uncertain (86). Sung and colleagues constructed a machine learning bloodstream infection classifier in pediatric patients receiving cancer treatments, demonstrating the feasibility of predictive analytics in this population (136).

The application of ML to microbiota data in ALL is still emerging but holds transformative potential.

Machine learning approaches-including random forests, support vector machines, and neural networks-have been applied to microbiota data for classification and prediction tasks (137). Emerging architectures such as transformers and graph neural networks show theoretical promise for capturing microbial network dynamics, though their application to clinical microbiota data remains in early stages.

### 8.4 Challenges in pediatric studies

Microbiome research in pediatric ALL faces distinctive methodological challenges that limit the generalizability and comparability of findings. Small sample sizes-reflecting the rarity of the disease, single-center study designs, and the logistical difficulties of biospecimen collection from pediatric patients-reduce statistical power and increase the risk of false-positive findings (138). Addressing the challenge of small sample sizes will require multi-center collaborative networks-such as the Children's Oncology Group (COG) and International Berlin-Frankfurt-Münster (iBFm) consortium-to standardize microbiome collection protocols and pool data across institutions. Prospective meta-analyses of standardized cohorts, shared biobanking initiatives, and harmonized sequencing pipelines are essential next steps. International consortia should establish common data elements for pediatric ALL microbiome studies, including standardized time points for sample collection, antibiotic exposure recording, and clinical endpoint definitions. Confounding variables abound: antibiotic exposure, dietary changes, hospitalization environment, geographic location, and genetic background all influence microbiota composition and are difficult to control in observational studies.

Standardization of microbiome analysis is a critical unmet need. Variability in sample collection methods (stool vs. rectal swab), storage conditions, DNA extraction protocols, sequencing platforms, and bioinformatics pipelines can introduce technical artifacts that obscure true biological signals (130). The Microbiome Quality Control project and similar initiatives have highlighted the substantial inter-laboratory variation in microbiome profiling results, emphasizing the need for standardized protocols and inclusion of appropriate control samples (139).

Longitudinal study designs-with frequent sampling across treatment phases-are essential for capturing the temporal dynamics of treatment-induced dysbiosis but are resource-intensive and dependent on patient compliance (20, 22). The use of sibling controls-while practical-introduces shared household environment and genetic factors that may mask disease-specific microbiota differences. Population-based healthy controls matched for age, geography, diet, and antibiotic history would strengthen causal inference but are logistically challenging to recruit.

Despite these challenges, the field is maturing rapidly. International consortia, standardized protocols, and open-access bioinformatics tools are facilitating larger, more rigorous studies. The integration of microbiome endpoints into prospective clinical trials-coupled with careful metadata collection and multi-omic profiling-will accelerate the translation of microbiome science into clinical practice for children with leukemia.

## 9 Knowledge gaps and future directions

### 9.1 Knowledge gaps

A central methodological limitation confronting the microbiome-leukemia field concerns the inability of cross-sectional and case-control designs to differentiate causal contributions from incidental associations (17). The prevailing observational evidence characteristically documents compositional divergence between ALL patient cohorts and healthy comparator groups, yet such study architectures are inherently incapable of determining whether detected dysbiotic patterns actively promote leukemogenesis, arise secondarily from malignancy-associated alterations in host metabolism and immune function, or simply mirror concurrent antibiotic exposure, dietary modification, and hospitalization effects (140). Resolution of these competing explanations will necessitate prospective birth cohort investigations with serial microbiota sampling commencing in the neonatal period and extending through the window of peak leukemia incidence, complemented by rigorously controlled preclinical studies employing gnotobiotic and antibiotic manipulation approaches to establish whether specific microbial configurations influence leukemia initiation kinetics or disease progression trajectories. (Figure 4).

**Figure 4:**
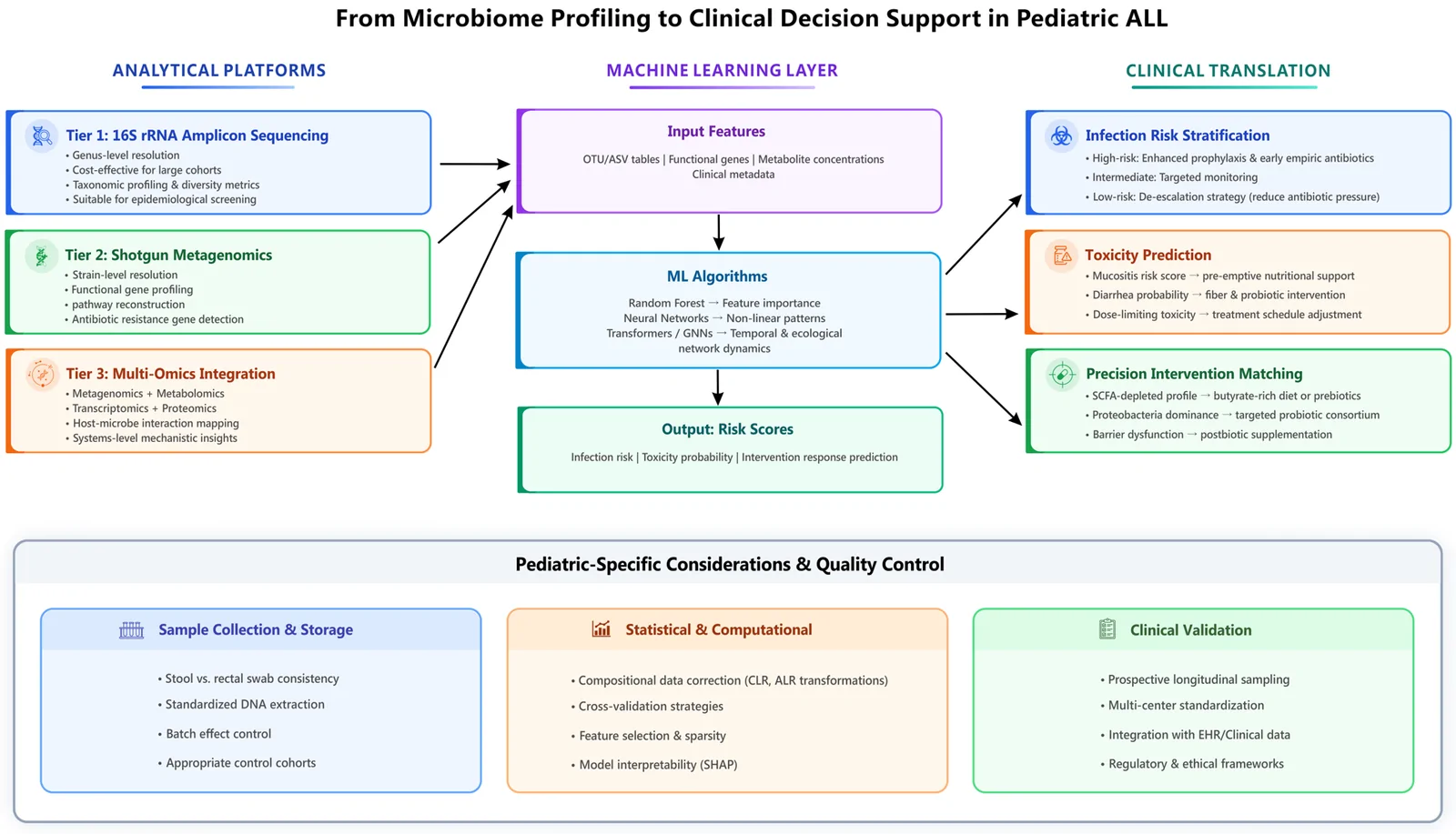
Multi-omics integration framework for precision supportive care in pediatric ALL. Tiered analytical platforms (16S rRNA, shotgun metagenomics, multi-omics) feed into machine learning algorithms (Random Forest, neural networks, transformers/GNNs) to generate infection risk, toxicity probability, and intervention response scores. Clinical translation encompasses infection risk stratification, toxicity prediction, and precision intervention matching. The bottom panel highlights pediatric-specific considerations in sample collection, statistical computation, and clinical validation.

The germ-free and gnotobiotic mouse models developed by Greaves and colleagues provide a powerful framework for testing causal hypotheses (41, 44). ETV6-RUNX1-transgenic mice raised under germ-free conditions fail to develop leukemia until exposed to specific pathogens or microbial products, directly implicating microbiota-driven immune modulation in disease pathogenesis (44). Extension of these models to examine the effects of specific bacterial consortia, SCFA supplementation, or dietary interventions on leukemia risk would yield mechanistic insights with translational relevance. Complementary approaches using humanized microbiota models-where germ-free mice are colonized with human fecal microbiota from ALL patients or healthy donors-could bridge the gap between murine and human biology, enabling preclinical testing of nutritional and microbiota-targeted interventions in a physiologically relevant context.

### 9.2 Future directions in pediatric ALL microbiome research

The extant literature derives primarily from individual institutions enrolling relatively small patient cohorts and employing non-standardized analytical protocols. Methodologically rigorous investigations spanning multiple pediatric oncology centers, following participants longitudinally with pre-specified specimen collection schedules, and adhering to harmonized procedures for biospecimen handling, nucleic acid extraction, and computational analysis are critically required to corroborate preliminary microbial signatures, pinpoint robust and generalizable predictive indicators, and adequately control for geographical, nutritional, and treatment-protocol sources of heterogeneity (138). Optimally, such studies would implement dense temporal sampling-ideally at weekly or fortnightly intervals-during the initial months following diagnosis, a period coinciding with maximal vulnerability to infectious complications and the most pronounced microbiota perturbations, thereby generating clinically actionable temporal resolution.

International consortia-such as the Children's Oncology Group (COG) and the International Childhood Acute Lymphoblastic Leukaemia Consortium-provide existing infrastructures that could support embedded microbiome studies within prospective clinical trials (141). The inclusion of microbiome endpoints in interventional trials would enable assessment of treatment effects on microbial composition and the impact of microbiota-targeted interventions on clinical outcomes.

### 9.3 Methodological advances

The lack of standardized methods limits comparability across studies and hampers meta-analytic synthesis (130). Harmonization of sample collection (fresh stool vs. preservative-based stabilization), DNA extraction protocols, sequencing platforms, and bioinformatics pipelines is essential for advancing the field. Reference standards, positive and negative controls, and inter-laboratory calibration should be routine components of microbiome studies. In preparing this review, we have consulted the the STORMS (Strengthening The Organization and Reporting of Microbiome Studies) consortium guidelines for reporting human microbiome research (142). While STORMS was designed primarily for primary research, we have endeavored to apply its principles of transparent reporting-including clear literature search methodology, evidence-level annotation, and standardized nomenclature-wherever applicable to this narrative review.

### 9.4 Clinical translation and implementation challenges

The inter-individual variability observed in microbiota composition and treatment response suggests that one-size-fits-all interventions may be suboptimal. Precision microbiota medicine-tailoring interventions based on individual baseline microbiota profiles, predicted metabolic capacity, and clinical risk factors-is an aspirational goal (143). Patients with severe baseline dysbiosis might benefit from FMT or defined consortia, while those with modest perturbations might respond to prebiotic or probiotic supplementation. Patients predicted to be at high risk for infectious complications based on microbiota signatures could receive enhanced prophylaxis or closer monitoring.

The development of such personalized approaches requires robust predictive models validated in independent cohorts, as well as randomized trials comparing stratified intervention strategies. Collaborative networks linking pediatric oncology centers with microbiota research institutions will be essential for generating the large, well-characterized datasets necessary to train and validate these predictive models. Machine learning models integrating microbiota data with clinical variables show promise for risk stratification, as discussed in Section 8, but require external validation and prospective testing before clinical implementation (86, 87) (Figure 4).

### 9.5 Integration with precision nutrition

The convergence of microbiota science and nutritional oncology offers a particularly promising avenue for pediatric ALL. Precision nutrition-the tailoring of dietary recommendations based on individual metabolic, microbiota, and genetic profiles-could optimize treatment tolerance and long-term health outcomes (143). By leveraging microbiota profiling to identify specific metabolic deficits-such as depleted SCFA production or impaired amino acid metabolism-clinicians could design personalized dietary prescriptions that address individual needs rather than applying generic nutritional guidelines. For patients with depleted SCFA producers, high-fiber or resistant starch interventions might restore metabolic function. For patients with impaired tryptophan metabolism, dietary tryptophan modulation or specific probiotic strains might support immune reconstitution.

The practical implementation of precision nutrition in pediatric oncology faces challenges: food preferences, treatment-related symptoms, family resources, and cultural dietary patterns all influence feasibility. Digital health tools-mobile applications for dietary tracking, personalized nutrition recommendations, and telehealth support-may facilitate adherence. Collaborative care models integrating oncology teams with registered dietitians and microbiota specialists will be essential for translating precision nutrition from concept to clinical reality.

The incorporation of microbiota endpoints into routine clinical care will require the development of rapid, cost-effective diagnostic tools. Current microbiome profiling methods-16S sequencing and shotgun metagenomics-remain too slow and expensive for real-time clinical decision-making. The development of targeted qPCR panels, biosensors, or simplified sequencing platforms capable of delivering actionable microbiota information within hours rather than days would transform the clinical utility of microbiota assessment in pediatric oncology. Point-of-care testing for key microbial biomarkers-such as Enterococcaceae dominance or SCFA-producing taxa abundance-could enable dynamic risk stratification and guide time-sensitive nutritional interventions during critical treatment windows.

Beyond scientific and regulatory challenges, the clinical implementation of microbiome-based interventions faces practical barriers that must be addressed. Cost: Microbiome profiling and customized probiotic formulations are currently expensive and rarely reimbursed by insurance. Accessibility: Specialized microbiome laboratories are concentrated in academic centers, limiting access for community hospitals. Patient acceptance: Families may hesitate to administer live microorganisms to immunocompromised children, requiring dedicated education and counseling. Workflow integration: Incorporating microbiome sampling and analysis into busy pediatric oncology clinics requires streamlined protocols, rapid turnaround times, and electronic health record integration. Addressing these barriers will be essential for translating microbiome science into routine clinical practice.

## 10 Conclusions

The intestinal microbiota occupies a uniquely central position at the interface of nutritional science, immunology, and pediatric oncology, presenting genuine prospects for meaningfully improving clinical outcomes in childhood leukemia. The present manuscript has integrated available evidence establishing that microbiota perturbations represent a pervasive feature of the pediatric ALL disease course-detectable at initial presentation, amplified during cytotoxic therapy, and demonstrable even years following treatment cessation. Available evidence suggests that these ecological changes may transcend passive bystander status, potentially participating in shaping leukemia biology, modulating treatment tolerance, and influencing survivor health trajectories through diverse mechanistic avenues encompassing immunomodulatory crosstalk, bioactive metabolite-mediated signaling, maintenance of gastrointestinal barrier competence, and epigenetic regulatory mechanisms.

The clinical implications are substantial. Microbiota composition shows preliminary predictive value for infectious complications during chemotherapy-the leading cause of treatment-related morbidity and mortality in pediatric ALL. Baseline enrichment in *Proteobacteria* predicts febrile neutropenia; *Enterococcaceae* and *Streptococcaceae* dominance is associated with subsequent infections; and depletion of beneficial taxa including *Bifidobacterium longum* and *Faecalibacterium prausnitzii* identifies patients at potentially elevated risk. These predictive associations raise the prospect of microbiota-guided risk stratification, enabling personalized infection prevention strategies. Although preliminary evidence suggests that microbiota composition is associated with infectious complications during chemotherapy, with baseline *Bifidobacterium longum* abundance showing modest predictive accuracy (AUC = 0.720, 95% CI not reported in original study; sensitivity and specificity not provided) in one study (23), whether microbiota-based prediction models can achieve clinically actionable accuracy requires validation in independent, multicenter cohorts.

Nutritional and microbiota-targeted interventions offer a particularly attractive therapeutic avenue for this vulnerable population. Prebiotics, select probiotics, enteral nutrition, and microbiota-supportive dietary patterns show promise for preserving microbial diversity, enhancing SCFA production, and maintaining intestinal barrier integrity during chemotherapy. The safety profile of prebiotics and certain probiotic strains in immunocompromised hosts appears favorable, though rigorous pediatric trials are needed to establish efficacy and optimal dosing. Fecal microbiota transplantation remains investigational but may have a role in refractory cases within clinical trial settings.

Looking forward, the integration of multi-omic profiling, machine learning, and precision nutrition heralds a new era of microbiota-informed oncology care. Standardized, multi-center longitudinal studies with embedded interventional trials are essential to validate microbiota biomarkers and test microbiota-targeted therapies. The translation of these advances into clinical practice will require interdisciplinary collaboration among oncologists, microbiologists, immunologists, nutrition scientists, and data analysts.

The economic and public health implications of microbiota-centered care in pediatric ALL are substantial. If microbiota-targeted nutritional interventions can reduce infectious complications by even a modest margin, the savings in hospitalization costs, antibiotic utilization, and treatment delays would be considerable. In resource-limited settings-where access to intensive supportive care and novel therapeutics is constrained-low-cost nutritional strategies to support microbiota health may offer a practical and scalable approach to improving outcomes. The development of affordable, culturally acceptable prebiotic and probiotic interventions tailored to regional dietary patterns and microbial ecologies should be prioritized in global pediatric oncology research agendas.

As we refine our understanding of the microbiota-leukemia axis, we move closer to a future where the gut microbiota is routinely assessed, monitored, and modulated as an integral component of comprehensive pediatric leukemia care. By nourishing the microbial partners that inhabit our patients' intestines, we may enhance treatment tolerance, reduce complications, and improve the long-term health of children battling leukemia. The gut microbiota is not merely a bystander in childhood leukemia-it is a critical modulator, a predictive biomarker, and a therapeutic target whose potential is only beginning to be realized. The integration of microbiota science with precision nutrition offers a compelling pathway toward more personalized, effective, and compassionate care for the most vulnerable of cancer patients.
